# In Situ Polymerization on a 3D Ceramic Framework of Composite Solid Electrolytes for Room‐Temperature Solid‐State Batteries

**DOI:** 10.1002/advs.202207744

**Published:** 2023-05-18

**Authors:** An‐Giang Nguyen, Rakesh Verma, Geon‐Chang Song, Jaekook Kim, Chan‐Jin Park

**Affiliations:** ^1^ School of Materials Science and Engineering Chonnam National University 77 Yongbong‐ro, Buk‐gu Gwangju 61186 South Korea; ^2^ Department of Chemistry University of Allahabad Prayagraj 211002 India

**Keywords:** 3D ceramic framework, composite solid electrolytes, in situ polymerization, solid‐state lithium batteries, solid‐state sodium batteries

## Abstract

Solid‐state batteries (SSBs) are ideal candidates for next‐generation high‐energy‐density batteries in the Battery of Things era. Unfortunately, SSB application is limited by their poor ionic conductivity and electrode‐electrolyte interfacial compatibility. Herein, in situ composite solid electrolytes (CSEs) are fabricated by infusing vinyl ethylene carbonate monomer into a 3D ceramic framework to address these challenges. The unique and integrated structure of CSEs generates inorganic, polymer, and continuous inorganic–polymer interphase pathways that accelerate ion transportation, as revealed by solid‐state nuclear magnetic resonance (SSNMR) analysis. In addition, the mechanism and activation energy of Li^+^ transportation are studied and visualized by performing density functional theory calculations. Furthermore, the monomer solution can penetrate and polymerize in situ to form an excellent ionic conductor network inside the cathode structure. This concept is successfully applied to both solid‐state lithium and sodium batteries. The Li|CSE|LiNi_0.8_Co_0.1_Mn_0.1_O_2_ cell fabricated herein delivers a specific discharge capacity of 118.8 mAh g^−1^ after 230 cycles at 0.5 C and 30 °C. Meanwhile, the Na|CSE|Na_3_Mg_0.05_V_1.95_(PO_4_)_3_@C cell fabricated herein maintains its cycling stability over 3000 cycles at 2 C and 30 °C with zero‐fading. The proposed integrated strategy provides a new perspective for designing fast ionic conductor electrolytes to boost high‐energy solid‐state batteries.

## Graphical abstract

The monomer is injected into and polymerized inside the 3D inorganic framework to form a 3D composite solid electrolyte that can promote ion transportation through inorganic, polymer, and continuous inorganic–polymer interphase pathways, as revealed by NMR analysis. To demonstrate the practical application of this approach, the fabricated cells can power light‐emitting diodes and 2‐wheel‐drive toy cars.

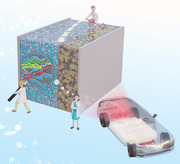

## 1 Introduction

Beyond the Internet of Things era, in which all products are connected to the Internet, the Battery of Things era, in which all products are connected to batteries, has emerged. Nowadays, wireless connections are rapidly replacing wired connections in diverse products ranging from tiny devices such as earphones and smart pens to electric vehicles (EVs). In the Battery of Things era, lithium‐ion batteries (LIBs) will serve as the heart of all electronic devices and widely revolutionize our lifestyle, in addition to facilitating decarbonization from a sustainability perspective.^[^ ^1^ ^]^ Unfortunately, the energy density and safety concerns of conventional LIBs that employ liquid organic electrolytes limit their use in future applications.^[^ ^2^ ^]^ Therefore, solid‐state batteries (SSBs) are becoming more appealing as an ideal candidate for next‐generation batteries to address these limitations.^[^ ^1^ ^]^ In this scenario, SSBs can potentially revive the “holy grail” of metallic lithium metal anode with the highest specific capacity (3860 mAh g^−1^), lowest metallic density (0.59 g cm^−3^), and strongest negative electrochemical potential (−3.04 V vs SHE).^[^ ^3^ ^]^ Furthermore, as a critical component, solid‐state electrolytes (SSEs) affect the performance of SSBs.^[^ ^1^, ^2^ ^]^ Therefore, the highly safe and high‐energy‐density SSBs could be realized through the successful development of SSEs.

Generally, SSEs can be divided into three categories, namely inorganic solid electrolytes (ISEs), solid polymer electrolytes (SPEs), and composite solid electrolytes (CSEs).^[^ ^4^ ^]^ ISEs, such as garnet–type Li_5_La_3_M_2_O_12_ (M = Nb, Ta, Zr),^[^ ^5^ ^]^ NaSICON–type Li_1+_ *_x_* Al*_x_* Ti_2−_ *_x_* (PO_4_)_3_,^[^ ^6^ ^]^ and Li_1+_ *_x_* Al_y_Ge_2−_ *_y_* (PO_4_)_3_,^[^ ^7^ ^]^ perovskite‐type Li_3_ *_x_* La_2/3−_ *_x_* TiO_3_,^[^ ^8^ ^]^ sulfide,^[^ ^9^ ^]^ and halide,^[^ ^10^ ^]^ possess high mechanical strength and can suppress the growth of lithium dendrites. However, stack pressure or the addition of liquid electrolytes is needed to reduce the interfacial resistance between rigid ISEs and electrodes.^[^ ^11^ ^]^ By contrast, SPEs, such as poly(ethylene oxide),^[^ ^12^ ^]^ poly(propylene carbonate),^[^ ^13^ ^]^ poly(acrylonitrile),^[^ ^14^ ^]^ poly(vinylidene fluoride) (PVDF),^[^ ^15^ ^]^ and poly(methyl methacrylate) (PMMA),^[^ ^16^ ^]^ are flexible, have excellent processability, and provide good interfacial contact with electrodes.^[^ ^4b^ ^]^ Nonetheless, their ionic conductivity and mechanical properties are generally unsatisfactory.^[^ ^2b^ ^]^ Because they inherit the advantages of both SPEs and ISEs, CSEs are promising candidate SSEs with acceptable ionic conductivity and good contact with electrodes.^[^ ^2^, ^17^ ^]^

As is well known, CSEs are constructed by dispersing (in)active fillers into the polymer matrix to facilitate the dissociation of lithium salts, disorder the crystallization of the polymer matrix, and subsequently, increase the ionic conductivity of CSEs.^[^ ^18^ ^]^ To further improve the ionic conductivity of CSEs, fillers of various morphologies such as 0D, 1D, 2D, and 3D frameworks are being investigated to improve the conductive pathways for lithium‐ion diffusion.^[^ ^2^, ^18^ ^]^ Among them, 3D frameworks have significantly improved ionic conductivity by constructing “highways” for lithium‐ion diffusion.^[^ ^2^, ^4^ ^]^ In addition, 3D frameworks can eliminate the risk of filler particle aggregation and prohibit the growth of lithium dendrites.^[^ ^4a^ ^]^ To date, several strategies for preparing 3D (in)active frameworks have been investigated, including the hydrogel method,^[^ ^19^ ^]^ template method (e.g., PMMA and NaCl),^[^ ^20^ ^]^ and 3D printing.^[^ ^21^ ^]^ Among them, the template methods are simple and most attractive. However, PMMA is an expensive template material, and if NaCl is used, an additional washing step is required to remove it.^[^ ^20c,f^ ^]^ Therefore, a more effective template method is required for fabricating 3D frameworks.

In the meantime, many CSEs have been fabricated using the tape‐casting method, which requires the use of a solvent.^[^ ^2^, ^4^ ^]^ When the solvent is used, a subsequent drying process and a solvent recovery process are required, and even after drying, residual solvent species may affect the electrochemical properties of CSEs.^[^ ^22^ ^]^ Furthermore, the interphase between electrodes and CSE may be non‐conformal. To alleviate this problem, in situ polymerization has been explored as a novel and meaningful strategy.^[^ ^23^ ^]^ During battery assembly, monomer solutions are injected, and then, the polymerization reaction occurs to form SPEs.^[^ ^24^ ^]^ Accordingly, integration of the electrode and electrolyte and fast ionic transport through the interface is achieved. Furthermore, liquid monomers can be permeated in the pores of the cathode layer to construct a potentially excellent ionic conducting network.^[^ ^25^ ^]^

In response to the scenarios described above, a novel in situ CSE for high‐performance SSBs for use at room temperature is synthesized in this study. The in situ 3D Li_6.4_La_3_Zr_1.4_Ta_0.6_O_12_ CSE (3D‐LLZT‐CSE) with 3D ion transport pathways is fabricated by injecting a solution containing vinylene ethylene carbonate (VEC) monomer into the 3D‐LLZT framework, which was prepared with the tape casting method by using starch powder as a template material, and subsequently, polymerizing the VEC monomer. As mentioned above, the excellent electrolyte/electrode interface is integrated through in situ polymerization, and the 3D‐LLZT framework can promote the fast and continuous formation of lithium‐ion conducting pathways while improving the conductivity of the inorganic–polymer interface. The in situ 3D‐LLZT‐CSE exhibits an exceptional ionic conductivity of 0.8036 mS cm^−1^ at 30 °C. Owing to these synergetic effects, the solid‐state lithium battery (SSLB) composed of Li|3D‐LLZT‐CSE|LiNi_0.8_Co_0.1_Mn_0.1_O_2_ delivers a specific discharge capacity of 118.8 mAh g^−1^ after 230 cycles at 0.5 C, corresponds to a capacity retention of 79.4% at 30 °C. Furthermore, by using 3D‐Na_3.3_Zr_1.7_La_0.3_(SiO_4_)_2_(PO_4_) (3D‐NZLSP) as a framework, this innovative approach is successfully applied to a solid‐state sodium battery (SSSB). The resulting Na|3D‐NSLSP‐CSE|Na_3_Mg_0.05_V_1.95_(PO_4_)_3_@C cell exhibits a reversible capacity of 95.0 mAh g^−1^ after 3000 cycles at a rate of 2 C with zero‐fading.

## 2 Results and Discussion

### 2.1 Solid‐State Lithium Batteries

#### 2.1.1 Material Characterization

**Figure 1:**
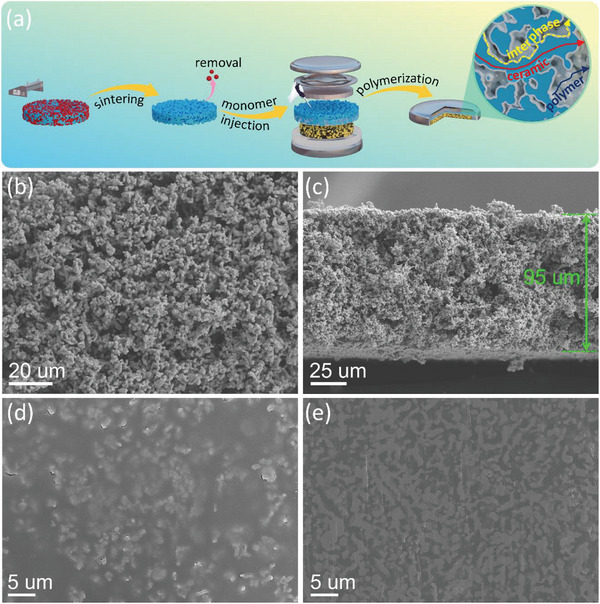
a) Schematic diagram of the fabrication of 3D‐LLZT‐CSE. b) Top and c) cross‐sectional SEM images of 3D‐LLZT. d) Top and e) cross‐sectional SEM images of 3D‐LLZT‐CSE.

The 3D‐LLZT‐CSE synthesis process is described in detail in the experiment section (Supporting Information) and illustrated in **Figure** **1**a. Previous studies have demonstrated that garnet‐type LLZT materials exhibit high Li^+^ conductivity (≈10^−3^ S cm^−1^ at room temperature), an electrochemical stability window of up to ≈6 V versus Li/Li^+^, and considerable chemical stability against Li metal.^[^ ^26^ ^]^ Consequently, LLZT was selected as the ceramic component for this investigation. Furthermore, VEC was employed as the starting monomer due to the presence of a C=C bond in its vinyl group, which allows for polymerization. Additionally, the five‐membered ring containing the C=O double bond can attenuate the intermolecular interactions between poly VEC (PVEC) polymer chains, as will be discussed subsequently. Therefore, PVEC can facilitate ion transport to realize high ionic conductivity. In brief, the 3D‐LLZT framework was prepared by heating it at 1100 °C for 1 h to remove the starch template and other organic components. Then, 10 µL of the monomer‐containing solution was dropped on both the cathode and the Li anode. Thereafter, the 3D‐LLZT framework was placed on the top of the cathode, after the monomer was injected to completely fill the pores of the 3D‐LLZT. Finally, the entire cell was heated at 70 °C for 10 h to complete the polymerization reaction and form an integrated cathode‐supported electrolyte structure.

The porous 3D‐LLZT framework was observed by scanning electron microscopy (SEM). As shown in Figure 1b,c, a 3D continuous porous network structure with a thickness of ≈95 µm was successfully synthesized. Moreover, according to the top‐view and cross‐sectional SEM images of the 3D‐LLZT‐CSE after its in situ polymerization, as shown in Figure 1d,e, the polymer can evenly occupy the pores of the 3D‐LLZT framework and generate continuous pore‐free structures, which is extraordinarily significant for the construction of continuous Li^+^ transport channels.

**Figure 2:**
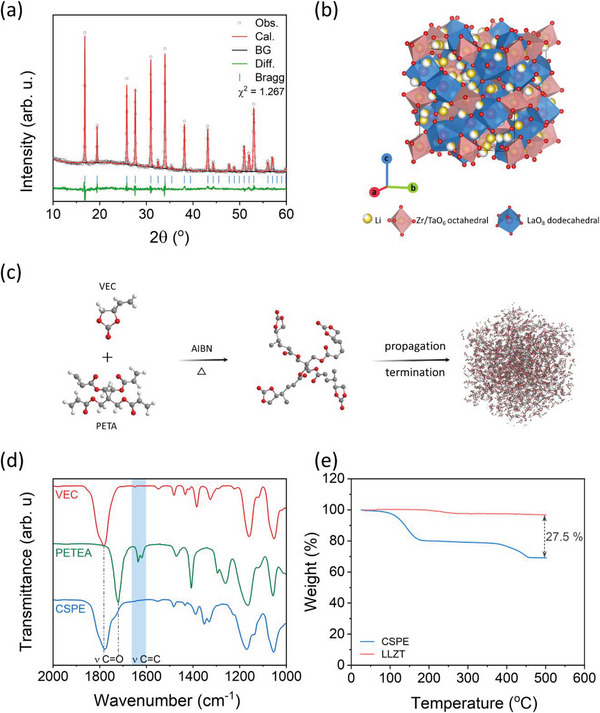
a) Rietveld refinement on XRD pattern and b) crystal structure of 3D‐LLZT. c) Polymerization mechanism of monomer. d) FTIR spectra of VEC, PETA, and 3D‐LLZT‐CSE. e) TGA curves of 3D‐LLZT and 3D‐LLZT‐CSE.

The crystallographic structures of the 3D‐LLZT sample were analyzed using XRD. The diffraction patterns, including the Rietveld refinement of 3D‐LLZT, are presented in **Figure** **2**a,b. The obtained diffraction peaks of 3D‐LLZT were well indexed to a cubic structure with the $Ia\bar{3}d$ space group. In the unit cell, La^3+^ occupies the 8‐fold coordinated 24c position, Ta^4+^ and Zr^4+^ are located at the octahedrally coordinated 16a position, the Li is presented at the 24 d and 94 h sites, and the O existed at the 96 h sites, as shown in Figure 2b and Table S1, Supporting Information. The cubic structure of LLZT is known to promote lithium conductivity at room temperature.^[^ ^26^ ^]^ Additionally, a comparison of the XRD patterns of LLZT powder and 3D‐LLZT (Figure S2a, Supporting Information) revealed that all diffraction peaks were well‐matched without any impurity phase (COD #96‐155‐2157). This implies that the cubic phase was maintained even during high‐temperature sintering.^[^ ^27^ ^]^ BET analysis was also carried out (Figure S2b, Supporting Information). The 3D‐LLZT exhibited a specific surface area of 1.87 m^2^ g^−1^ and a pore volume of 0.018 cm^3^ g^−1^, with an average pore diameter of 38.4 nm. Furthermore, the 3D‐LLZT framework retained its flat film structure, but its diameter decreased marginally to 18 mm (Figure S3a, Supporting Information).

Figure 2c shows the polymerization reaction mechanism of VEC. The reaction was initiated by azobisisobutyronitrile (AIBN), which releases free radicals upon heating and attacks the vinyl group of VEC to promote PVEC formation. FTIR analysis of the VEC monomer, pentaerythritol tetraacrylate (PETA) crosslinker, and 3D‐LLZT‐CSE were performed to confirm the polymerization mechanism (Figure 2d). The C=C stretching peak located at 1650 cm^−1^ originating from VEC and the peak at 1634 cm^−1^ from PETA disappeared after polymerization. This indicated that the VEC monomer and PETA were converted to a cross‐linking polymer after heating at 70 °C for 10 h. In addition, the transparent liquid solution of LiTFSI, PETA, and VEC monomer turned into a translucent solid polymer (Figure S3b, Supporting Information). Furthermore, the conversion percentage of VEC in the monomer solution can be determined from the integrated area ratio of CH_2_ = in the monomers in SPE relative to that in the monomer solution, based on the 1H NMR spectra depicted in Figure S3c, Supporting Information.^[^ ^28^ ^]^ Consequently, the conversion percentage of VEC to PVEC was found to be 59.03%. Additionally, a new peak corresponding to hydrogen in saturated carbon at 1.45 ppm was detected. Furthermore, the dispersion of PETEA peaks suggests its complete polymerization. The molecular weight of SPE (Mn: 45363, PDI: 4.144) was also verified through gel permeation chromatography, as shown in Figure S4a, Supporting Information. To determine the amount of polymer and LLZT in CSE, TGA was performed (Figure 2e). The TGA curve of 3D‐LLZT‐CSE has two sloping regions, one from 30 to 200 °C corresponding to the decomposition of PVEC and the other from 300 to 500 °C corresponding to the decomposition of LiTFSI and PETA (Figure S4b, Supporting Information). The total loss of 3D‐LLZT‐CSE was 30.9 wt.%. By contrast, 3D‐LLZT remained almost stable with only 3.4 wt.% loss up to 500 °C. The difference in weight loss percentages between 3D‐LLZT and 3D‐LLZT‐CSE by 27.5 wt% was ascribed to the presence of organic compounds in CSE, and the remaining 72.5 wt.% can be assigned to the inorganic components of CSE. Additionally, the 3D‐LLZT‐CSE was thermally stable up to 110 °C, and the resulting weight loss was less than 3 wt.%.

#### 2.1.2 Electrochemical Analysis

**Figure 3:**
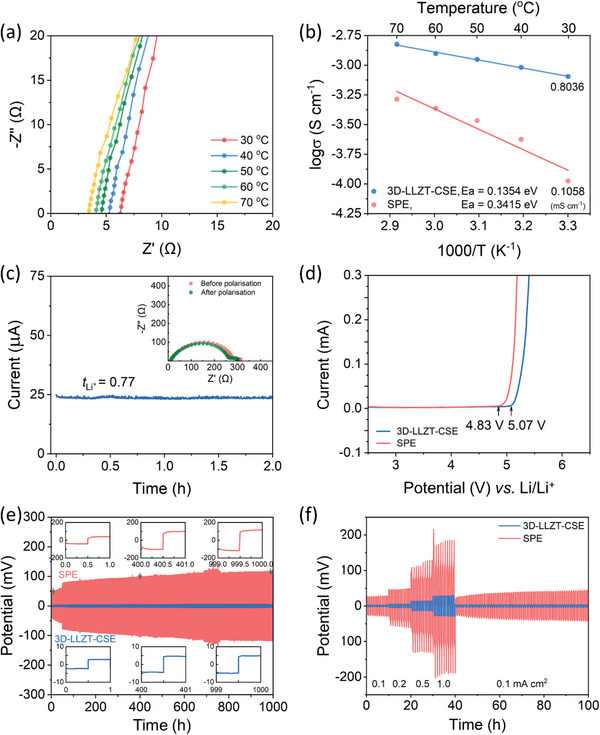
a) Nyquist plots recorded at different temperatures. b) Arrhenius plots depicting conductivity. c) Current transient profile and corresponding EIS plots of Li|3D‐LLZT‐CSE|Li symmetric cell before and after polarization. d) Linear sweep voltammogram of SPE and 3D‐LLZT‐CSE. e) Cyclability at a current density of 0.1 mA cm^−2^ and f) rate capabilities of Li|SPE and 3D‐LLZT‐CSE|Li symmetric cells at 30 °C.

The Li^+^ conductivity (*σ*) of the electrolytes was studied in the temperature range of 30–70 °C by means of electrochemical impedance spectroscopy (EIS). The component fraction of a mixture of starch powder as a pore‐formation agent and LiTFSI salt was investigated systematically by using the EIS technique to optimize the ionic conductivity of the 3D‐LLZT‐CSE, while the concentrations of FEC and PETA were fixed. The ratio of VEC and LiTFSI was optimized at the mole ratio of 9. Further, by using the ratio, the highest ionic conductivity of 3D‐LLZT‐CSE (0.8036 mS cm^−1^ at 30 °C) was achieved with 20 wt.% of starch powder (Figure S5, Supporting Information). Furthermore, based on the results in **Figure** **3**a and Figure S6a, Supporting Information, the calculated ionic conductivity of SPE was 0.1058 mS cm^−1^ at 30 °C, which is considerably lower than the value of 0.8036 mS cm^−1^ of 3D‐LLZT‐CSE at the same temperature (Figure 3b). In addition, the activation energies (*E_a_*) of SPE and 3D‐LLZT‐CSE were found to be 0.3415 and 0.1354 eV, respectively. This improvement in the ionic conductivity and activation energy of 3D‐LLZT‐CSE was caused by Li^+^ transport through well‐aligned polymer matrices as well as the long‐range continuous Li^+^ diffusion pathways in the 3D‐LLZT backbone and the solid–polymer interphase.

Lithium transference number $\left(t_{Li^{+}}\right)$ is another important parameter to be considered for the evaluation of electrolytes. We used the chronoamperometry technique to investigate $t_{Li^{+}}$ by using lithium symmetric Li|SPE or 3D‐LLZT‐CSE|Li cells. The corresponding Nyquist plots before and after polarisation are shown in Figure 3c and Figure S6b, Supporting Information. The SPE has a high $t_{Li^{+}}$ of 0.6 at 30 °C because of the strong electron‐withdrawing effect of the C=O groups in PVEC that facilitates Li^+^ transportation.^[^ ^25c]^ Furthermore, the 3D‐LLZT‐CSE has an excellent $t_{Li^{+}}$ of 0.77 at 30 °C. This indicates that the presence of 3D‐LLZT can provide additional conducting paths for Li^+^ through continuous LLZT networks and the LLZT–polymer interphase. The $t_{Li^{+}}$ enhancement suggests that the highly conducting route, along with the LLZT network and its interphase with the polymer, plays a crucial role in accelerating Li^+^ diffusion.

In addition, the electrochemical stability potential of the electrolytes is a vital parameter to evaluate electrolytes. Therefore, the asymmetric Li|SPE or 3D‐LLZT‐CSE|stainless steel cells were subjected to linear sweep voltammetry (LSV) test at a scan rate of 1 mV s^−1^ at 30 °C. According to LSV results in Figure 3d, the 3D‐LLZT‐CSE was stable up to 5.07 V versus Li/Li^+^, while the SPE started to decompose above 4.83 V. The increased electrochemical stability potential of the former can be ascribed to the high LLZT content in the 3D‐LLZT‐CSE.

To assess the compatibility of the 3D‐LLZT‐CSE with Li metal, a symmetric Li|3D‐LLZT‐CSE|Li cell was assembled for galvanostatic Li plating/stripping testing at 30 °C. Figure 3e presents the long‐term Li stripping/plating profiles of the Li|3D‐LLZT‐CSE|Li cell at a current density of 0.1 mA cm^−2^, with selected cyclic potential profiles displayed in the inset of Figure 3e. The Li|3D‐LLZT‐CSE|Li cell exhibited flat profiles and outstanding stability over 1000 h without failure. Furthermore, the overpotential values of the Li|3D‐LLZT‐CSE|Li cell were minimal, increasing marginally from 2.7 mV initially to 4.8 mV after 1000 h. In contrast, the Li|SPE|Li symmetric cell demonstrated a large overpotential during Li plating/stripping, indicating the superior stability of the 3D‐LLZT‐CSE in contact with Li metal. The stability of the 3D‐LLZT‐CSE against Li metal was further examined under various current densities, ranging from 0.1 to 1.0 mA cm^−2^ for each of the 10 cycles at 30 °C, as shown in Figure 3f. The overpotentials of the Li|3D‐LLZT‐CSE|Li symmetric cell corresponding to current densities of 0.1, 0.2, 0.5, and 1.0 mA cm^−2^ were 2.6, 5.4, 12.9, and 28.0 mV, respectively. Additionally, when the current density decreased to 0.1 mV cm^−2^, the Li|3D‐LLZT‐CSE|Li cell maintained the original square wave‐like potential profiles with a small overpotential of 3.3 mV. In contrast, the Li|SPE|Li cell exhibited much larger polarization compared to the Li|3D‐LLZT‐CSE|Li cell and lost the square‐wave‐like potential profiles at high current density. Furthermore, the Li|3D‐LLZT‐CSE|Li symmetric cell demonstrated outstanding plating/stripping performance at a higher current density of 1 mA cm^−2^, sustaining over 500 h of operation (Figure S6c, Supporting Information). These results emphasize that the 3D‐LLZT‐CSE can effectively suppress lithium dendrite growth, suggesting that the proposed CSE possesses novel properties.

**Figure 4:**
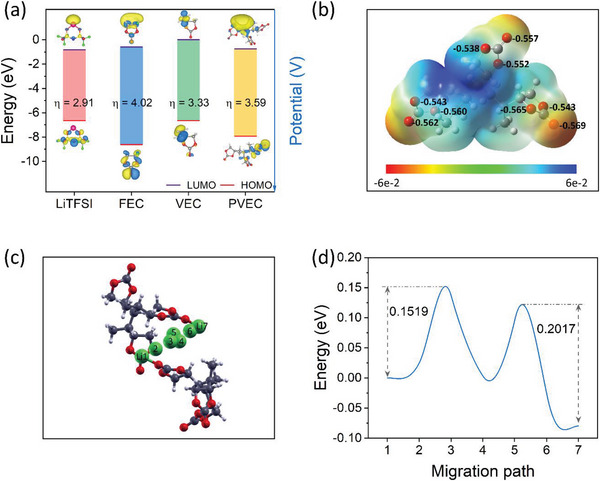
a) LUMO and HOMO energy values of LiTFSI, FEC, VEC, and PVEC. b) Electrostatic potential maps and natural bond orbital (NBO) charge of PVEC. c) Optimum Li^+^ migration pathway between two PVEC chains, and d) the corresponding Li^+^ activation energy barrier from the Li1 to Li7 sites.

Density functional theory (DFT) was used to clarify the reduction and oxidation behaviors of the electrolyte components. According to the frontier molecular orbital theory, in the highest occupied molecular orbital (HOMO), the electrons located in the outer orbitals with high energy tend to donate electrons easily, and therefore, they are associated with lower oxidation potential stability. By contrast, in the lowest unoccupied molecular orbital (LUMO), the inner unoccupied orbitals with low energy have strong electron affinity, which is correlated with the reduction in the higher‐potential region. As shown in **Figure** **4**a, after the VEC monomer was polymerized to PVEC, the HOMO level shifted to the lower‐energy region, from −6.646 eV for VEC to −7.916 eV for PVEC, indicating that PVEC can be stable in the higher‐potential region. In addition, the HOMO levels were ranked in the following order: LiTFSI > PVEC > FEC. The LUMO levels were ranked in the following order: LiTFSI > PVEC > FEC. Moreover, chemical hardness (*η*), which reflects the relative reactivity of the molecule, was calculated as the mean of the energy gap between HOMO and LUMO. A low *η* implies that the molecule is reactive and decomposes easily.^[^ ^29^ ^]^ As shown in Figure 4a, LiTFSI had the lowest *η* of 2.91 eV. Accordingly, LiTFSI tends to decompose preferentially to form the interphase layer, as will be discussed later in the XPS analysis section. By contrast, FEC, which has the lowest HOMO, can enhance the stability of the high‐voltage cathode.

To further study the Li^+^ transportation mechanism in PVEC, the electrostatic potential maps and natural bond orbital (NBO) charge of PVEC with a repeat unit of 3 were calculated. As shown in Figure 4b, the surface was colored blue for positive electrostatic potential, which attracts anion, and red for negative electrostatic potential, which attracts Li^+^ cations. Moreover, the oxygen atoms in the carbonyl groups (C=O) had NBO charges of −0.557, −0.569, and −0.562 owing to the electron‐withdrawing effect. These values were marginally lower than those of the oxygen atoms of O–C–O with NBO charges of (−0.538, −0.552), (−0.543, −0.565), and (−0.543, −0.562), respectively. Moreover, steric hindrance refers to the physical obstruction imposed by the five‐membered ring in the VEC, which impedes Li^+^ mobility. The electrostatic potential maps depicted in Figure 4b reveal that the increased positive charge on the five‐membered ring of VEC could repel Li^+^, consequently attenuating the interaction between the C−O groups and Li^+^. This, in turn, promotes the interaction between C = O and Li^+^.^[^ ^23^, ^25^ ^]^ TFSI^–^ can be captured along the carbon chain with a positive NBO charge, which improves the lithium transference number. Therefore, Li^+^ migrates through PVEC mainly owing to interaction with the oxygen atom of the C=O group. Furthermore, during Li^+^ migration, the highest energy along the diffusion pathway is the activation energy *E_a_* for this process. Therefore, the activation energy of Li^+^ was determined using the nudged elastic band (NEB) method, as shown in Figure 4c,d and Supporting Video S1, Supporting Information. A low activation energy *E_a_* of 0.1519 and 0.2017 eV for forward and backward migration paths was obtained, respectively. Significantly, the *E_a_* value is lower than that derived from the Arrhenius plot (Figure 3b), primarily due to the exclusion of the effects of other components (e.g., TFSI^−^, PETA, FEC) in the analysis. Nevertheless, this lower *E_a_* value could theoretically contribute to enhanced ionic conductivity and superior electrochemical properties, while simultaneously promoting accelerated reaction kinetics.

The outstanding properties of 3D‐LLZT‐CSE suggest that this 3D composite is a promising electrolyte for use in high‐energy‐density solid‐state lithium batteries (SSLBs). Additionally, as a cathode material, the Ni‐rich LiNi_0.8_Co_0.1_Mn_0.1_O_2_ (NCM811) material is promising, because it can provide high energy density and is inexpensive. Despite these merits, Ni‐rich cathodes suffer from structural instability and parasitic reactions during cycling, sometimes limiting commercialization. In this study, to mitigate these concerns, NCM811 triple‐doped with Ti, Zr, and Al was used as the cathode material. The NCM811 cathode was characterized by XRD, XPS, and SEM. As shown in Figure S7a,b, Supporting Information, Rietveld refinements of the XRD patterns indicated that NCM811 exhibited the O3‐type layered (hexagonal *α*‐NaFeO_2_) structure with the $R\bar{3}m$ space group and did not contain any impurity (Table S2, Supporting Information). This result indicated that elemental Ti, Zr, and Al were satisfactorily incorporated into the NCM811 structure. Moreover, the characteristic peaks of Ti 2p, Zr 3d, and Al 2p were detected by XPS, thus confirming the presence of Ti, Zr, and Al, as depicted in Figure S7 c–f, Supporting Information. Moreover, as revealed by the SEM image in Figure S8, Supporting Information, NCM811 had a sphere‐like shape, and the size of the secondary particles was ≈15 µm.

**Figure 5:**
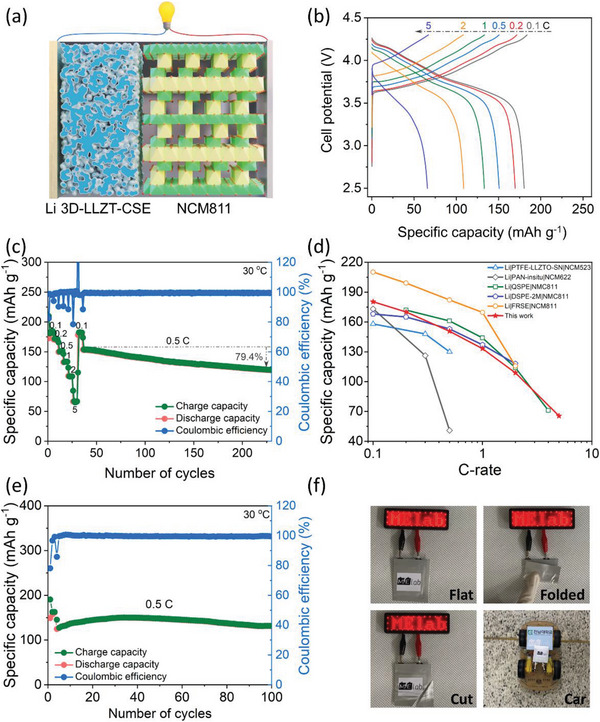
a) Schematic of Li|3D‐LLZT‐CSE|NCM811 cell. b) Galvanostatic charge‐discharge profiles, and c) corresponding rate capability of Li|3D‐LLZT‐CSE|NCM811 cell at 30 °C. d) Rate capability performance comparison of various solid electrolyte‐based cells using NCM cathode at ambient temperature.^[^ ^14^, ^25^, ^30^ ^]^ e) Cylability of Li|3D‐LLZT‐CSE|NCM811 pouch cell at 30 °C. f) Photographs of Li|3D‐LLZT‐CSE|NCM811 pouch cell that lights up the LED in the flat, folded, cut conditions, and powers a 2‐wheel‐drive toy car.

Furthermore, the performance of the NCM811 cathode was pre‐evaluated in a half‐cell by using 1 M LiPF_6_ in EC/DEC liquid electrolyte. As depicted in Figure S9a,b, Supporting Information, NCM811 exhibited outstanding performance, and its specific discharge capacities were 193.4, 186.1, 175.7, 165.3, 148.4, 113.9, and 53.9 mAh g^−1^ at the C‐rates of 0.1, 0.2, 0.5, 1, 2, 5, and 10 C, respectively. These results confirmed the synergistic effect of the Al‐, Zr‐, and Ti‐doped NCM811, and this material may achieve satisfactory electrochemical properties in SSLBs. Therefore, an SSLB was configured with the above‐described NCM811 cathode material, as illustrated in **Figure** **5**a. The Li|3D‐LLZT‐CSE|NCM811 cell delivered initial charge and discharge capacities of 209.7 and 172.7 mAh g^−1^, respectively, corresponding to an initial CE of 82.3% at the rate of 0.1 C at 30 °C, as depicted in Figure 5b,c. Moreover, it delivered specific discharge capacities of 180.3, 170.0, 150.7, 133.2, 108.8, and 65.5 mAh g^−1^ at the C‐rates of 0.1, 0.2, 0.5, 1, 2, and 5 C, respectively. When the C‐rate returned to 0.1 C, the Li|3D‐LLZT‐CSE|NCM811 cell recovered to approximately its initial stage with a specific discharge capacity of 178.9 mAh g^−1^. Even after the cell was cycled 230 times consecutively at 0.5 C, it delivered a specific discharge capacity of 118.8 mAh g^−1^, which corresponds to a capacity retention of 79.4% with an average Coulombic efficiency of 99.43% (Figure 5c). In contrast, the Li|SPE|NCM811 delivered a low specific capacity of 10 mAh g^−1^ at 0.1 C due to its low ionic conductivity at 30 °C (Figure S9c, Supporting Information). Furthermore, the performance of the Li|3D‐LLZT‐CSE|NCM811 cell was comparable to that of recently reported cells using an NCM cathode with solid‐state electrolytes at ambient temperature (Figure 5d and Table S3, Supporting Information).^[^ ^14^, ^25^, ^30^ ^0]^

Additionally, a pouch‐type Li|3D‐LLZT‐CSE|NCM811 cell was fabricated and tested. After three cycles at 0.1 C, the pouch cell delivered an average discharge capacity of 131.0 mAh g^−1^ over 100 cycles at 0.5 C (Figure 5e). Moreover, to demonstrate the feasibility of the SSLBs, the Li|3D‐LLZT‐CSE|NCM811 pouch cell successfully operated the light‐emitting diodes (LEDs) circuit for ≈1.7 min even under folded or cut conditions (Figure 5f, and Figure S10 and Video S2, Supporting Information). Then, unfortunately, this pouch cell could not provide sufficient energy to restart this complicated LED circuit. Therefore, it was connected with a simple “CNU” logo composed of 23 red LEDs in a parallel array and still lit up 23 LEDs for 20 min under high humidity of 69% even after the second cut (Figure S10 and Video S2, Supporting Information). The above results clearly verify the great flexibility and safety of SSLBs. To highlight the practical application of Li|3D‐LLZT‐CSE|NCM811, the series connection of six pouch cells drove a 2‐wheel‐drive toy car ≈200 m smoothly for ≈15 min (Figure 5f and VideoS3, Supporting Information). This implies that the Li|3D‐LLZT‐CSE|NCM811 combination is a potential candidate for high‐performance SSLBs.

**Figure 6:**
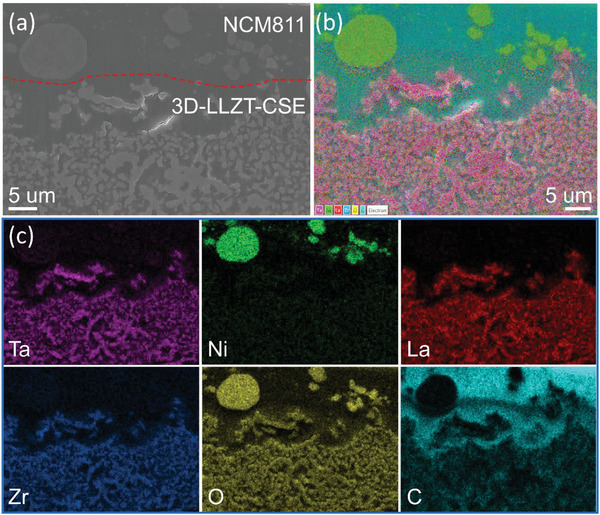
a) SEM image and b,c) corresponding EDS maps of the interface between the NCM811 cathode and 3D‐LLZT‐CSE.

**Figure 7:**
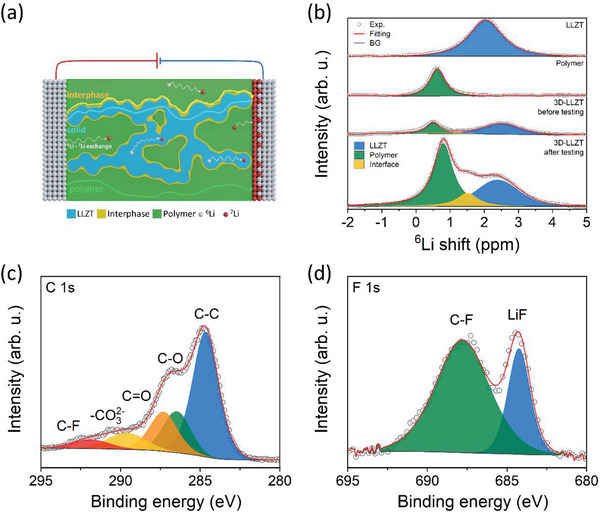
a) Schematic representation of the principle of the ^6^Li tracer‐exchange process and Li^+^ conduction pathway in 3D‐CSE. b) ^6^Li SSNMR spectra of LLZT and 3D‐LLZT‐CSE before and after polarization. High‐resolution XPS spectra of c) C 1s and d) F 1s of Li electrode after cycling test.

To reveal the excellent performance of 3D‐LLZT‐CSE, the cross sections of the NCM811 cathode and 3D‐LLZT‐CSE were checked using SEM (**Figure** **6**). For comparison, the cross‐section of the bare NCM811 cathode was examined using SEM, and it was found to have a highly porous structure (Figure S11, Supporting Information). As shown in Figure 6, all of the pores in both the NCM811 cathode and the 3D‐LLZT were successfully filled and integrated by the in situ polymer. The integrated structure can promote and guarantee fast charge transfer, thus ensuring that the SSLB works well. Furthermore, to identify the roles of each component of CSE, magic‐angle spinning solid‐state nuclear magnetic resonance (MAS‐SSNMR) analysis of ^6^Li was performed for 3D‐LLZT‐CSE, while the polymer and LLZT powder were used as reference samples. In nature, lithium has two stable isotopes, namely, ^6^Li and ^7^Li, with natural abundances of 7.5% and 92.5% on Earth, respectively. By applying an external potential to the ^6^Li|3D‐LLZT‐CSE|^6^Li symmetric cell, ^6^Li^+^ passes through and partially replaces ^7^Li^+^ in CSE. Therefore, the active components of CSE are enriched with ^6^Li owing to the tracer‐exchange process, as illustrated in **Figure** **7**a. The Li^+^ conducting pathways encoded with specialized chemical information in CSE can be identified by comparing the ^6^Li evolution of each of the components before and after polarization. The ^6^Li chemical shifts of LLZT and SPE occurred at 2.03 and 0.61 ppm, respectively, as shown in Figure 7b. In the case of 3D‐LLZT‐CSE, before testing, the areas under the fitting curves of LLZT, polymer, and their interphase were quantified as 1350.10, 731.61, and 105.72, respectively (Table S4, Supporting Information). After testing, the corresponding areas evolved to 3793.19, 5950.05, and 1333.89, increasing by factors of 2.8, 8.1, and 12.6, respectively. Based on this result, the percentage contributions of LLZT, polymer, and the LLZT–polymer interphase on ionic conductivity were 34.22%, 53.69%, and 12.09%, respectively. The remarkable improvements in the increase factor and contribution percentage of the polymer phase indicate that the aligned polymer network in the 3D‐LLZT‐CSE serves as the most favorable Li^+^ migration pathway. Moreover, the interphase between LLZT and polymer is a helpful pathway for Li^+^ transportation, apart from the continuous LLZT phase. Consequently, the significant improvement in Li^+^ diffusion, which led to the high‐performance 3D‐LLZT‐CSE, can be ascribed to the PVEC polymer, continuous LLZT network, and their interphase.

Additionally, XPS analysis was conducted to analyze the SEI composition of the Li anode after cycling. As shown in Figure 7c, the C 1s characteristic peak was deconvoluted into five peaks at 284.7, 286.5, 287.3, 289.6, and 291.9 eV corresponding to the C−C, C−O, C=O, $CO_{3}^{2-}$, and C−F bonds (of –CF_3_ groups in TFSI^‐^), respectively. Furthermore, the F 1s peak was deconvoluted into two peaks at 684.3 and 687.8 eV corresponding to the LiF and C−F components, respectively, as shown in Figure 7d. According to previous reports, the LiF‐rich SEI layer can prohibit the continuous decomposition of electrolytes and suppress the growth of lithium dendrites.^[^ ^25^, ^31^ ^]^ This multicomponent SEI can improve electrochemical properties, provide good electronic insulation, and ensure the long‐term stability of the anode interphase.

### 2.2 Solid‐State Sodium Batteries

#### 2.2.1 Material Characterization

**Figure 8:**
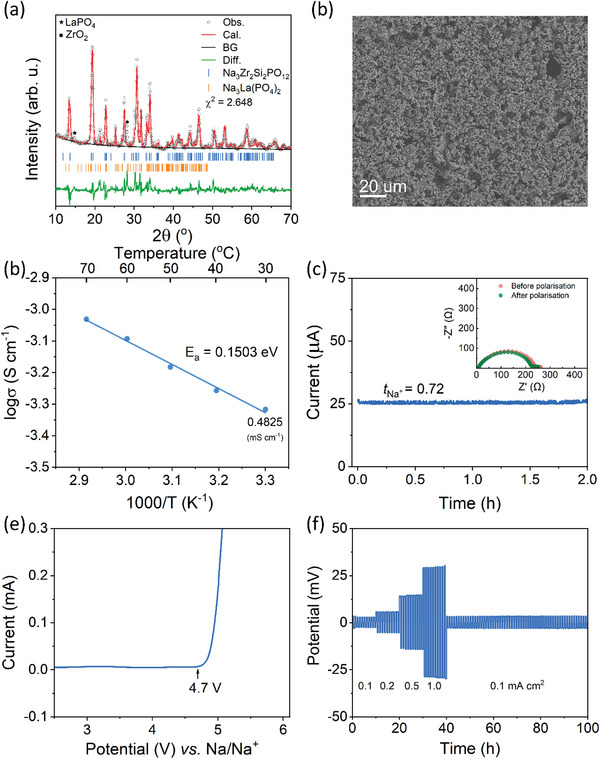
a) Rietveld‐refined XRD pattern and b) SEM image of 3D‐NZLSP. c) Arrhenius plots of conductivity. d) Current transient profile and the corresponding EIS plots of the Na|3D‐NZLSP‐CSE|Na symmetric cell before and after polarization. e) Linear sweep voltammogram of 3D‐NZLSP‐CSE. f) Rate capabilities of Na|3D‐NZLSP‐CSE|Na symmetric cell at 30 °C.

Beyond LIBs, other rechargeable batteries such as lithium‐sulfur batteries (LSBs), sodium‐ion batteries (SIBs), and potassium‐ion batteries (PIBs), are attractive for further reducing the cost of batteries and potentially replacing LIBs in the near future.^[^ ^32^ ^]^ Among them, SIBs have several advantages such as the abundance of sodium resources on Earth and sodium being the next element in IA with physicochemical properties similar to those of lithium.^[^ ^33^ ^]^ For these reasons, the development of SIB technology may be accelerated, and it is expected that SIBs will be commercialized soon in the Battery of Things era. In this context, is it possible to apply the 3D‐CSE concept to SSSBs? To answer this question, in this study, we synthesized Na_3.3_Zr_1.7_La_0.3_(SiO_4_)_2_(PO_4_) (NZLSP) by following the sol‐gel method and used it as the starting inorganic material to prepare 3D‐CSE.^[^ ^34^ ^]^ NZLSP has a NaSICON‐type structure with excellent ionic conductivity, as well as chemical and thermal stability; it was discovered by Goodenough et al.^[^ ^35^ ^]^ XRD analysis was performed to confirm the crystal structure of the as‐prepared NZLSP (**Figure** **8**a and Figure S12a, Supporting Information). NZLSP exhibited the C2/c space group consisting of a SiO_4_/PO_4_ tetrahedral sharing corner and a ZrO_6_ octahedron (Figure S12b and Table S5, Supporting Information). Interestingly, the La dopant self‐formed a new phase composed of Na_3_La(PO_4_)_2_ instead of being incorporated into the NaSICON structure. In addition, low‐intensity peaks of LaPO_4_ and ZrO_2_ at 14.59° and 28.23°, respectively, were detected. According to a previous report, the Na_3_La(PO_4_)_2_ phase can increase the concentration of Na^+^ ions in the NaSICON main phase to improve ionic conductivity.^[^ ^34^ ^]^ The 3D‐NZLSP framework exhibited a highly porous morphology, as demonstrated by SEM images in Figure 8b.

#### 2.2.2 Electrochemical Analysis

The *σ* of 3D‐NZLSP‐CSE was studied in the temperature range of 30–70 °C by means of EIS (Figure 8c and Figure S13, Supporting Information). The calculated *σ* of 3D‐NZLSP‐CSE increased synchronously as the temperature increased, and it was 0.4825 mS cm^−1^ at 30 °C. Moreover, the *E_a_* of 3D‐NZLSP‐CSE can be determined by fitting the temperature‐dependent *σ* plot to the Arrhenius equation, as explained in the Supporting Information. The resulting low *E_a_* value of 0.1503 eV implies the existence of a low energy barrier for Na^+^ diffusion in CSE. In addition to the superior *σ*, 3D‐NZLSP‐CSE exhibited a high Na^+^ transference number of 0.72 (Figure 8d) and was stable up to 4.7 V versus Na/Na^+^, as measured by means of LSV (Figure 8e). This extraordinary nature of 3D‐NZLSP‐CSE can be ascribed to the synergistic effect of the polymer, 3D‐NZLSP, and their interphase, as explained previously for 3D‐LLZT‐CSE.

In addition, Na plating/striping was performed at various current densities of 0.1, 0.2, 0.5, and 1.0 mA cm^−2^ in each of the 10 cycles at 30 °C to investigate the stability of CSE against the Na electrode, as shown in Figure 8f. The Na|3D‐NZLSP‐CSE|Na symmetric cell exhibited flat potential profiles with small overpotentials of ≈3.1, 5.9, 14.5, and 29.9 mV, respectively. Moreover, the symmetric cell maintained a similar potential profile with a small overpotential of 3.6 mV when the current density returned to 0.1 mV cm^−2^. This result, too, verifies the superior stability of 3D‐NZLSP‐CSE with the Na electrode.

Considering the strong electrochemical properties of 3D‐NZLSP‐CSE, the electrolyte was further evaluated in cells configured with the as‐synthesized Na_3_Mg_0.05_V_1.95_(PO_4_)_3_@C (NVMP@C) as a cathode material with long cyclability and high stability. Magnesium was selected as a lightweight dopant to enhance the ionic and electronic conductivity of Na_3_V_2_(PO_4_)_3_ material. According to the XRD pattern of the NVMP@C cathode (Figure S14a, Supporting Information), all peaks can be well indexed to the NaSICON structure with the $R\bar{3}c$ space group.^[^ ^36^ ^]^ This structure is composed of Na(1) at the 6b and Na(2) at the 18e sites in a 3D open VO_6_ octahedral structure interlinked with a PO_4_ tetrahedral structure (Figure S14b and Table S6, Supporting Information), which favors rapid Na^+^ diffusion. For this reason, NVMP is a promising cathode for use in SIBs. Moreover, NVMP was coated with 5.7 wt.% of a graphitic‐like carbon material to further improve its electronic conductivity (Figure S14c,d), and it had a highly porous structure, as characterized by SEM (Figure S15, Supporting Information). Additionally, XPS was used to confirm the successful synthesis of NVMP@C, and it revealed the presence of Mg 1s, V^3+^, and V^4+^ peaks in the V 2p spectra (Figure S16, Supporting Information). Owing to the different valence states of V^3+^ and Mg^2+^, the V^4+^ state was newly formed to maintain charge neutrality.

**Figure 9:**
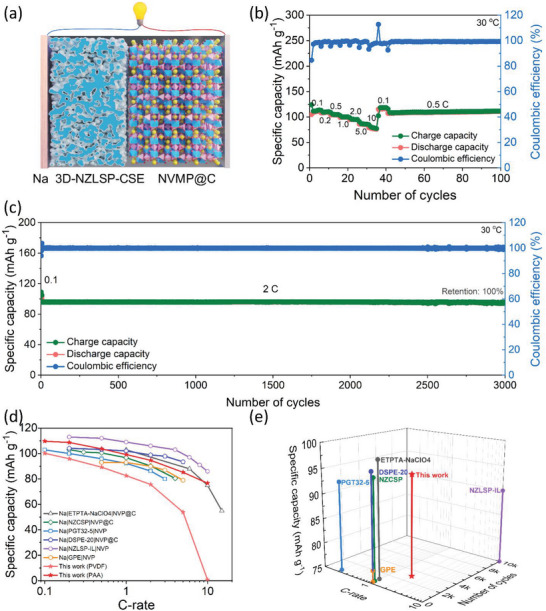
a) Schematic of the Na|3D‐NZLSP‐CSE|NVMP@C cell. b) Galvanostatic charge‐discharge profiles and c) the corresponding rate capability of the Na|3D‐NZLSP‐CSE|NVMP@C cell at 30 °C. d) Rate capability and e) cyclability performance comparison of various solid electrolyte cells that use NaSICON cathodes at ambient temperature.^[^ ^34^, ^37^ ^]^

Furthermore, NVMP@C based electrode was fabricated using NVMP@C active material, Super P carbon, and polyacrylic acid (PAA) binder in a weight ratio of 7:2:1. The NVMP@C electrode was pre‐tested in liquid electrolyte composed of 0.6 M NaPF_6_ in EC/DMC (30:70 in vol.%) with 5 vol.% FEC, and it exhibited high discharge capacities of 112.8, 112.3, 110.9, 109.3, 106.4, 110.9, 94.3 and 86.7 mAh g^−1^ at the rates of 0.1, 0.2, 0.5, 1.0, 2.0, 5.0, 10, and 20 C, respectively (Figure S17, Supporting Information). These results suggest that the NVMP@C electrode was successfully synthesized and may have good electrochemical properties when used in SSSBs. For this reason, an SSSB was constructed using a Na metallic anode, the 3D‐NZLSP‐CSE electrolyte, and the NVMP@C cathode, as depicted in **Figure** **9**a. The rate performance of the Na|3D‐NZLSP‐CSE|NVMP@C cell was investigated, and the results are presented in Figure 9b. This cell delivered an initial charge capacity of 123.9 mAh g^−1^ and a discharge capacity of 104.9 mAh g^−1^ at the rate of 0.1 C, which corresponds to an initial Coulombic efficiency of 84.6%. In addition, the reversible discharge capacities were 109.7, 108.7, 103.7, 99.2, 94.7, 85.3, and 76.7 mAh g^−1^ at the rates of 0.1, 0.2, 0.5, 1.0, 2.0, 5.0, and 10 C, respectively. When the C‐rate was turned back to 0.1 and 0.5 C, the original discharge capacity could be recovered (Figure 9b). In the long‐term cycling test, the Na|3D‐NZLSP‐CSE|NVMP@C cell delivered a reversible capacity of 95.0 mAh g^−1^ after 3000 cycles at a rate of 2 C with zero‐fading, as well as an average Coulombic efficiency of ≈100.0% (Figure 9c).

Note that the PAA binder is a strong self‐associated polymer and is widely used for preparing both cathodes and anodes.^[^ ^32^ ^]^ To explore the effect of the binder on the performance of the Na|3D‐NZLSP‐CSE|NVMP@C cell, another cell was fabricated under the same conditions, except conventional polyvinylidene fluoride (PVDF) was used as the binder instead of PAA. A comparison of the rate capabilities of both cells is shown in Figure S18a, Supporting Information. The discharge capacities of the SSSB fabricated using the PVDF binder were 100.2, 95.9, 89.3, 82.6, 75.7, 53.9, and 0.7 mAh g^−1^ at the rates of 0.1, 0.2, 0.5, 1.0, 2.0, 5.0, and 10 C, respectively. Interestingly, the cell retained similar discharge capacities when the C‐rate was changed back to 0.1 and 0.5 C. Nevertheless, the SSSB fabricated using the PAA binder displayed higher specific capacities and less polarization over various C‐rates than those of the cells fabricated using the PVDF binder (Figure S18b,c, Supporting Information). The electrochemical performance of the NVMP@C cathode in SSSBs was improved significantly owing to the strong adhesion property of PAA, which helped to maintain good contact between each electrode component. The outstanding rate capability and cycling performance of the Na|3D‐NZLSP‐CSE|NVMP@C cell were achieved owing to the synergistic effect of the high ionic conductivity of 3D‐NZLSP‐CSE and the high adhesion strength of the PAA binder. To the best of our knowledge, the cyclability and rate capability of the Na|3D‐NZLSP‐CSE|NVMP@C cell in this study are comparable or even superior to the values reported in recent studies in solid electrolytes and the NaSICON (Na_3_V_2_(PO_4_)_3_) cathode at room temperature, as shown in Figure 9d,e and summarized in Table S7, Supporting Information.^[^ ^34^, ^37^ ^]^ In addition, a pouch type Na|3D‐NZLSP‐CSE|NVMP@C cell was also assembled and evaluated. As shown in Figure S18d, Supporting Information, this pouch cell delivered a discharge capacity of 73.2 mAh g^−1^ after 100 cycles at 1 C. Furthermore, this readily powered a “CNU” logo with 23 LEDs under various conditions of flat, folded, and cut for ≈10 min at high humidity of 64% (Figure S19 and Video S4, Supporting Information), which verified its flexibility and safety.

To determine the composition of the SEI layer and further understand the superior cycling stability of 3D‐NZLSP‐CSE, the cycled Na anode was characterized by XPS. The C 1 s spectra can be deconvoluted into C−C, C−O, C = O, $CO_{3}^{2-}$, and C−F bonding peaks at 284.7, 286.7, 288.9, 290.5, and 292.0 eV, respectively (Figure S20a, Supporting Information). Meanwhile, the F 1s peak was fitted to NaF and C−F at 683.5 and 687.8 eV, respectively, as shown in Figure S20b, Supporting Information. All of the deconvoluted spectra revealed that the SEI layer on the surface of Na metal mainly consisted of NaF and organic components, which further improved the stability and electrochemical properties of 3D‐NZLSP‐CSE.

## 3 Conclusion

The in situ 3D‐LLZT and 3D‐NSLSP CSEs reported herein were rationally designed and fabricated for solid‐state lithium batteries (SSLBs) and solid‐state sodium batteries (SSSBs), respectively, with excellent electrochemical performance. In the case of SSLBs, 3D‐LLZT‐CSE exhibited a high ionic conductivity of 0.8036 mS cm^−1^, high $t_{Li^{+}}$ of 0.77, and a wide electrochemical window up to 5.07 V versus Li/Li^+^ at 30 °C. Additionally, density functional theory calculation was performed to investigate the mechanism and activation energy of Li^+^ transportation, by interaction with the oxygen atom of the C=O group in PVEC. The Li|3D‐LLZT‐CSE|NCM811 cell could be operated at up to 5 C at 30 °C with a reversible specific capacity of 65.5 mAh g^−1^. Moreover, this cell still delivered a specific discharge capacity of 95.0 mAh g^−1^, after 230 cycles at 0.5 C, which corresponds to a capacity retention of 79.4% with an average Coulombic efficiency of 99.43%. Meanwhile, in the case of SSSBs, 3D‐NZLSP‐CSE exhibited a high ionic conductivity of 0.4825 mS cm^−1^, high $t_{Na^{+}}$ of 0.72, and stability up to 4.7 V versus Na/Na^+^ at 30 °C. The Na|3D‐NSLSP‐CSE|NVMP@C cell delivered outstanding rate capability and cycling stability. In detail, it delivered a specific capacity of 95.0 mAh g^−1^ after 3000 cycles at a rate of 2 C with zero‐fading, as well as an average Coulombic efficiency of ≈100.0% at 30 °C. Even at a high rate of 10 C, a reversible capacity of 76.7 mAh g^−1^ was achieved. This was ascribed to the synergistic effect of the 3D continuous framework that accelerated the ion transportation through the inorganic, polymer, and continuous inorganic–polymer interphases, as revealed by the SSNMR analysis. This approach provides a practical strategy to address the bottlenecks of CSE for facilitating the construction of safe and high‐energy solid‐state batteries.

## 4 Experimental Section

The Experimental Section is available in the Supporting Information.

## Conflict of Interest

The authors declare no conflict of interest.

## Supporting information

Supporting Information

Supplemental Video 1

Supplemental Video 2

Supplemental Video 3

Supplemental Video 4

## Data Availability

The data that support the findings of this study are available from the corresponding author upon reasonable request.

## References

[1] a) S. Randau , D. A. Weber , O. Kotz , R. Koerver , P. Braun , A. Weber , E. Ivers‐Tiffee , T. Adermann , J. Kulisch , W. G. Zeier , F. H. Richter , J. Janek , Nat. Energy 2020, 5, 259;

[2] a) S. Li , S. Q. Zhang , L. Shen , Q. Liu , J. B. Ma , W. Lv , Y. B. He , Q. H. Yang , Adv. Sci. 2020, 7, 1903088; 10.1002/advs.201903088 PMC7055568 32154083

[3] D. C. Lin , Y. Y. Liu , Y. Cui , Nat. Nanotechnol. 2017, 12, 194. 28265117 10.1038/nnano.2017.16

[4] a) T. T. Ye , L. H. Li , Y. Zhang , Adv. Funct. Mater. 2020, 30, 2000077;

[5] R. Murugan , V. Thangadurai , W. Weppner , Angew. Chem., Int. Ed. 2007, 46, 7778. 10.1002/anie.200701144 17803180

[6] B. Lang , B. Ziebarth , C. Elsasser , Chem. Mater. 2015, 27, 5040.

[7] B. J. Sung , P. N. Didwal , R. Verma , A. G. Nguyen , D. R. Chang , C. J. Park , Electrochim. Acta 2021, 392, 139007.

[8] a) H. T. T. Le , R. S. Kalubarme , D. T. Ngo , H. S. Jadhav , C. J. Park , J. Mater. Chem. A 2015, 3, 22421;

[9] a) S. X. Deng , Y. P. Sun , X. Li , Z. H. Ren , J. W. Liang , K. Doyle‐Davis , J. N. Liang , W. H. Li , M. N. Banis , Q. Sun , R. Y. Li , Y. F. Hu , H. Huang , L. Zhang , S. G. Lu , J. Luo , X. L. Sun , ACS Energy Lett. 2020, 5, 1243;

[10] a) T. Asano , A. Sakai , S. Ouchi , M. Sakaida , A. Miyazaki , S. Hasegawa , Adv. Mater. 2018, 30, 1803075; 10.1002/adma.201803075 30216562

[11] C. C. Fang , B. Y. Lu , G. Pawar , M. H. Zhang , D. Y. Cheng , S. R. Chen , M. Ceja , J. M. Doux , H. Musrock , M. Cai , B. Liaw , Y. S. Meng , Nat. Energy 2021, 6, 987.

[12] L. Chen , Y. T. Li , S. P. Li , L. Z. Fan , C. W. Nan , J. B. Goodenough , Nano Energy 2018, 46, 176.

[13] P. N. Didwal , R. Verma , A. G. Nguyen , H. V. Ramasamy , G. H. Lee , C. J. Park , Adv. Sci. 2022, 9, 2105448. 10.1002/advs.202105448 PMC9069196 35240003

[14] M. Yao , Q. Q. Ruan , T. H. Yu , H. T. Zhang , S. J. Zhang , Energy Storage Mater. 2022, 44, 93.

[15] M. H. Woo , P. N. Didwal , H. J. Kim , J. S. Lim , A. G. Nguyen , C. S. Jin , D. R. Chang , C. J. Park , Appl. Surf. Sci. 2021, 568, 150934.

[16] J. H. Chen , Z. Yang , G. H. Liu , C. Li , J. S. Yi , M. Fan , H. P. Tan , Z. H. Lu , C. L. Yang , Energy Storage Mater. 2020, 25, 305.

[17] Y. Zheng , Y. Z. Yao , J. H. Ou , M. Li , D. Luo , H. Z. Dou , Z. Q. Li , K. Amine , A. P. Yu , Z. W. Chen , Chem. Soc. Rev. 2020, 49, 8790. 33107869 10.1039/d0cs00305k

[18] a) S. Tang , W. Guo , Y. Z. Fu , Adv. Energy Mater. 2021, 11, 2000802;

[19] a) D. C. Lin , P. Y. Yuen , Y. Y. Liu , W. Liu , N. Liu , R. H. Dauskardt , Y. Cui , Adv. Mater. 2018, 30, 1802661; 10.1002/adma.201802661 29939433

[20] a) K. Fu , Y. H. Gong , G. T. Hitz , D. W. McOwen , Y. J. Li , S. M. Xu , Y. Wen , L. Zhang , C. W. Wang , G. Pastel , J. Q. Dai , B. Y. Liu , H. Xie , Y. G. Yao , E. D. Wachsman , L. B. Hu , Environ. Sci. 2017, 10, 1568;

[21] a) S. Zekoll , C. Marriner‐Edwards , A. K. O. Hekselman , J. Kasemchainan , C. Kuss , D. E. J. Armstrong , D. Y. Cai , R. J. Wallace , F. H. Richter , J. H. J. Thijssen , P. G. Bruce , Environ. Sci. 2018, 11, 185;

[22] G. Foran , D. Mankovsky , N. Verdier , D. Lepage , A. Prebe , D. Ayme‐Perrot , M. Dolle , iScience 2020, 23, 101597. 33205013 10.1016/j.isci.2020.101597 PMC7648137

[23] J. C. Chai , Z. H. Liu , J. Ma , J. Wang , X. C. Liu , H. S. Liu , J. J. Zhang , G. L. Cui , L. Q. Chen , Adv. Sci. 2017, 4, 1600377. 10.1002/advs.201600377 PMC5323859 28251055

[24] Y. Y. Yan , J. W. Ju , S. M. Dong , Y. T. Wang , L. Huang , L. F. Cui , F. Jiang , Q. L. Wang , Y. F. Zhang , G. L. Cui , Adv. Sci. 2021, 8, 2003887. 10.1002/advs.202003887 PMC8097327 33977057

[25] a) F. Q. Liu , W. P. Wang , Y. X. Yin , S. F. Zhang , J. L. Shi , L. Wang , X. D. Zhang , Y. Zheng , J. J. Zhou , L. Li , Y. G. Guo , Sci. Adv. 2018, 4, aat5383; 10.1126/sciadv.aat5383 PMC6173527 30310867

[26] S. Abouali , C. H. Yim , A. Merati , Y. Abu‐Lebdeh , V. Thangadurai , ACS Energy Lett. 2021, 6, 1920.

[27] K. Kataoka , J. Akimoto , J. Ceram. Soc. Jpn. 2019, 127, 521.

[28] J. R. Wu , X. S. Wang , Q. Liu , S. W. Wang , D. Zhou , F. Y. Kang , D. Shanmukaraj , M. Armand , T. Rojo , B. H. Li , G. X. Wang , Nat. Commun. 2021, 12, 5746. 34593799 10.1038/s41467-021-26073-6 PMC8484457

[29] H. Fujimoto , S. Satoh , J. Phys. Chem. 1994, 98, 1436.

[30] a) T. L. Jiang , P. G. He , G. X. Wang , Y. Shen , C. W. Nan , L. Z. Fan , Adv. Energy Mater. 2020, 10, 1903376;

[31] Q. J. Zhou , C. K. Fu , R. L. Li , X. Y. Zhang , B. X. Xie , Y. Z. Gao , G. P. Yin , P. J. Zuo , Chem. Eng. J. 2022, 437, 135419.

[32] A. G. Nguyen , H. T. T. Le , R. Verma , D. L. Vu , C. J. Park , Chem. Eng. J. 2022, 429, 132359.

[33] a) R. Usiskin , Y. X. Lu , J. Popovic , M. Law , P. Balaya , Y. S. Hu , J. Maier , Nat. Rev. Mater. 2021, 6, 1020;

[34] Z. Z. Zhang , Q. H. Zhang , J. A. Shi , Y. S. Chu , X. Q. Yu , K. Q. Xu , M. Y. Ge , H. F. Yan , W. J. Li , L. Gu , Y. S. Hu , H. Li , X. Q. Yang , L. Q. Chen , X. J. Huang , Adv. Energy Mater. 2017, 7, 1601196.

[35] J. B. Goodenough , H. Y.‐P. Hong , J. A. Kafalas , Mater. Res. Bull. 1976, 11, 203.

[36] H. Li , X. Q. Yu , Y. Bai , F. Wu , C. Wu , L. Y. Liu , X. Q. Yang , J. Mater. Chem. A 2015, 3, 9578.

[37] a) Y. Lu , J. A. Alonso , Q. Yi , L. Lu , Z. L. Wang , C. W. Sun , Adv. Energy Mater. 2019, 9, 1901205;

