# Supplementary material for: In Situ Polymerization on a 3D Ceramic Framework of Composite Solid Electrolytes for Room‐Temperature Solid‐State Batteries
Source: Adv Sci (Weinh). 2023 May 18;10(21):2207744. doi: 10.1002/advs.202207744 (PMC10375120; doi:10.1002/advs.202207744)
Supplement: Supplementary file 1 — Supporting Information [file ADVS-10-2207744-s002.pdf]

ADVANCED SCIENCE
Open Access

Supporting Information

for *Adv. Sci.*, DOI 10.1002/advs.202207744

In Situ Polymerization on a 3D Ceramic Framework of Composite Solid Electrolytes for Room-Temperature Solid-State Batteries

*An-Giang Nguyen, Rakesh Verma, Geon-Chang Song, Jaekook Kim and Chan-Jin Park**

Supporting Information

In-situ Polymerization on a 3D Ceramic Framework of Composite Solid Electrolytes for Room-Temperature Solid-State Batteries

*An-Giang Nguyen, Rakesh Verma, Geon-Chang Song, Jaekook-Kim, and Chan-Jin Park**

A. G. Nguyen, R. Verma, G. C. Song, J. Kim, C. J. Park
School of Materials Science and Engineering, Chonnam National University, 77 Yongbong-ro, Buk-gu, Gwangju 61186, South Korea
E-mail: parkcj@jnu.ac.kr

R. Verma
School of Materials Science and Engineering, Chonnam National University, 77 Yongbong-ro, Buk-gu, Gwangju 61186, South Korea
Department of Chemistry, University of Allahabad, Prayagraj, Uttar Pradesh 211002, India

This Supporting Information file includes:

1. Experimental S4

1.1. Chemicals S4

1.2. Synthesis of materials S4

1.3. Preparation of composite solid electrolytes (CSEs) S6

1.4. Material characterization S6

1.5. Electrochemical characterization S8

1.6. Computational method S10

2. Tables S4

Table S1. Rietveld refinement results for the nominal composition of 3D-LLZT framework S4

Table S2. Rietveld refinement results for nominal composition of NCM811 .... S5

Table S3. Comparison of the electrochemical performances of solid electrolytes when using NCM cathode at room temperature S6

Table S4. Quantification of Li^+ local environment in 3D-LLZT-CSE before and after polarisation S7

Table S5. Rietveld refinement results obtained for nominal composition of 3D-NZLSP.... S8

Table S6. Rietveld refinement results obtained for nominal composition of $Na_3V_{1.95}Mg_{0.05}(PO_4)_3$@C S10

Table S7. Comparison of electrochemical performances of solid electrolytes and NaSICON at room temperature S11

3. Supporting Videos S12

4. Figures S13

Figure S1. TGA curve of $ZrO(NO_3)_2.xH_2O$ in air atmosphere. S13

Figure S2. (a) XRD patterns of LLZT powder and 3D-LLZT framework. (b) BET analysis results for 3D-LLZT. The BJH plot of the pore-size distribution is shown in the inset. S14

Figure S3. (a) Photographs of the 3D-LLZT framework before and after sintering, and (b) photographs of the monomer solution before and after polymerization. (c) ^{1}H NMR spectra of VEC, PETA, and SPE. S15

Figure S4. (a) GPC result of SPE. (b) TGA curves of PETA and LiTFSI in air atmosphere. S16

Figure S5. Ionic conductivity of 3D-LLZT-CSE as a function of (a) VEC/LiTFSI mole ratio and (b) wt.% of starch at 30 °C. S17

Figure S6. (a) Nyquist plots for SPE at different temperatures. (b) Current transient profile and the corresponding EIS plots of Li|SPE|Li symmetric cell before and after polarization. (c) Cyclability of the Li|3D-LLZT-CSE|Li symmetric cell at a current density of 1.0 mA cm^{-2} and a temperature of 30 °C. S18

Figure S7. (a) Rietveld-refined XRD pattern and (b) crystal structure of NCM811. (c) Wide-range XPS spectrum and high-resolution XPS spectra of (d) Ti 2p, (e) Zr 3d, (f) Al 2p in NCM811 material. S19

Figure S8. SEM images of NCM811 material at (a) low and (b) high magnification. S20

Figure S9. (a) Cyclability curve at 0.5 C and (b) rate capability of NCM811 cathode when using 1 M $LiPF_6$ in EC/DEC (50:50 vol.%) as the liquid electrolyte. (c) Galvanostatic charge-discharge profiles of Li|SPE|NCM811 at 0.1 C. S21

Figure S10. Photographs of Li|3D-LLZT-CSE|NCM811 pouch cell that lights up the LED in the flat, folded, cut conditions with time. S22

Figure S11. (a) Cross-sectional SEM image and (b, c) EDS mapping image of bare NCM811 electrode S23

Figure S12. XRD patterns of NZLSP powder and 3D-NZLSP framework. (b) Crystal structure of 3D-NZLSP. S24

Figure S13. Nyquist plots of 3D-NZLSP-CSE at different temperatures. S25

Figure S14. (a) Rietveld-refined XRD pattern and (b) crystal structure. (c) TGA curve in air atmosphere. (d) Raman spectrum of NVMP@C cathode. S26

Figure S15. SEM image of NVMP@C material. S27

Figure S16. High-resolution XPS spectra of (a) Mg 1s and (b) V 2p of NVMP@C material. S28

Figure S17. (a) Rate capability and (b) corresponding galvanostatic charge-discharge profiles of NVMP@C cathode at different C-rates when using 0.6 M $NaPF_6$ in EC/DMC (30:70 vol.%) with 5 vol.% FEC additive as liquid electrolyte. S29

Figure S18. (a) Rate capability comparison of Na|3D-NZLSP-CSE|NVMP@C cell and (b, c) their corresponding galvanostatic charge-discharge profiles at different C-rates when using PAA and PVDF binders, respectively. (d) Cylability of Na|3D-NZLSP-CSE|NVMP@C pouch cell at 30 °C S30

Figure S19. Photographs of Na|3D-NZLSP-CSE|NVMP@C pouch cell that lights up the LED in the flat, folded, cut conditions with time. S31

Figure S20. High-resolution XPS spectra of (a) C 1s and (b) F 1s of Na electrode after cycling test. S32

References **S33**

1. Experimental

1.1. Chemicals

The following materials were purchased and used in this study. Lithium nitrate ($LiNO_3$), zirconium(IV) oxynitrate hydrate ($ZrO(NO_3)_2.xH_2O$), lanthanum(III) nitrate hexahydrate ($La(NO_3)_3.6H_2O$), sodium nitrate ($NaNO_3$), ammonium dihydrogen phosphate ($NH_4H_2PO_4$), citric acid, povidone (PVP), poly(vinyl butyral) (PVB), benzyl butyl phthalate (BBP), lithium bis(trifluoromethane)sulfonimide (LiTFSI), pentaerythritol tetraacrylate (PETA), fluoroethylene carbonate (FEC), magnesium acetate tetrahydrate ($Mg(CH_3COO)_2$), poly(acrylic acid) (PAA, M_w 450k), 1-Methylpyrrolidin-2-one (NMP), ethylene carbonate (EC), dimethyl carbonate (DMC), sodium hexafluorophosphate ($NaPF_6$), and sodium (Na) were purchased from Sigma-Aldrich. Vinyl ethylene carbonate (VEC) and sodium bis(trifluoromethylsulfonyl)imide (NaTFSI) were purchased from TCI, while tantalum pentachloride ($TaCl_5$), tetraethyl orthosilicate (TEOS), ammonium vanadium oxide (NH_4VO_3), and starch powder were acquired from Thermo Fisher Scientific. Polyvinylidene fluoride (PVDF, Mw 600k) was received from MTI Korea. Acetone, ethanol, sodium hydroxide (NaOH), azobisisobutyronitrile (AIBN), and ethylene glycol were purchased from Daejung, while starch powder (DIFCO) was ordered from Saehan. $LiNi_{0.8}Co_{0.1}Mn_{0.1}O_2$ (NCM811) cathode material was obtained from Umicore. All chemicals were used as received, except AIBN, which was recrystallized, and the degree of hydration of $ZrO(NO_3)_2.xH_2O$ was determined by performing TGA analysis before it was used, as shown in **Figure S1**.

1.2. Synthesis of materials

$Li_{6.4}La_3Zr_{1.4}Ta_{0.6}O_{12}$ (LLZT) powder was synthesized by following the sol-gel method.[1] Stoichiometric amounts of $LiNO_3$ (10 mol% excess), $La(NO_3)_3.6H_2O$, $ZrO(NO_3)_2.xH_2O$, and $TaCl_5$ were dissolved in 100 mL deionised water (DI water) containing citric acid and ethylene glycol (1:1 molar ratio). The number of moles of citric acid was double the total number of

moles of cations. This solution was stirred for several hours and evaporated at 80 °C; subsequently, it was transferred to a vacuum oven and fully dried at 80 °C. The obtained powder was treated at 400 °C for 10 h in air atmosphere and then ground to obtain a fine powder. The fine powder was calcined at 900 °C for 12 h in air atmosphere.

$Na_{3.3}Zr_{1.7}La_{0.3}(SiO_4)_2(PO_4)$ (NZLSP) particles were prepared by following a modified sol-gel method.[2] First, TEOS was dissolved in a mixture of DI water and ethanol in the molar ratio of 1:10:4. To this solution, citric acid was added under vigorous stirring at 80 °C. Second, 100 mL DI water containing stoichiometric amounts of $NaNO_3$, $ZrO(NO_3)_2.xH_2O$, and $La(NO_3)_3.6H_2O$ was added to the above solution. Third, a stoichiometric amount of 0.6 M $NH_4H_2PO_4$ in DI water was added to the previous solution under stirring for several hours at 80 °C to obtain collosol. Finally, the collosol was completely dried at 80 °C in a vacuum oven. Subsequently, the dried collosol was transferred to a crucible and heated at 500 °C for 5 h and 1000 °C for 24 h at a heating rate of 5 °C min^{-1} to obtain NZLSP. To compensate for the loss of Na and P at high annealing temperatures, an additional 10 mol% $NaNO_3$ and $NH_4H_2PO_4$ were used, and the number of moles of citric acid was double the total number of moles of cations.

$Na_3V_{1.95}Mg_{0.05}(PO_4)_3$@C (NVMP@C) was synthesized as described in a previous report.[3] In brief, stoichiometric amounts of NaOH (10 mol% excess), NH_4VO_3, $NH_4H_2PO_4$, $Mg(CH_3COO)_2$ were added to DI water containing a certain amount of citric acid. The number of moles of acid was equal to the number of moles of NH_4VO_3. The above solution was stirred for 4 h at 80 °C and then dried at 80 °C for 10 h in a vacuum oven to obtain a powder. Then, the powder was pretreated at 350 °C for 4 h in air atmosphere, followed by carbonization at 800 °C for 8 h in Ar:H_2 mixed atmosphere (95:5 vol%) inside a tube furnace.

All powders were stored in a vacuum desiccator for further use.

1.3. Preparation of composite solid electrolytes (CSEs)

LLZT, PVP as a surfactant, PVB as a binder, BBP as a plasticizer, and starch as a porous agent were mixed in a solution containing ethanol and acetone (5:5 in vol.%) in the following mass ratios: 100:5:10:20:20, respectively. Moreover, 10 wt.% of $LiNO_3$ was added to compensate for the loss of Li during the subsequent heat treatment step. The starch powder content can be varied to control the pore size of the porous layer. Thereafter, the slurry was cast on a nylon tape by using a doctor blade and dried at 80 °C. This tape was punched into circles measuring 19 mm in diameter, after which the nylon tape was removed. Then, the organic component was removed from the LLZT films by heating the above samples at 1100 °C for 1 h. The thickness of the final LLZT film was controlled to 100 μm.

NZLSP was prepared by following the same process, except NZLSP powder and $NaNO_3$ as precursors were used instead of LLZT powder and $LiNO_3$, respectively.

Electrolyte precursor solutions were prepared by dissolving the designed amount of LiTFSI or NaTFSI salt in 1 g of VEC monomer with 0.5 wt.% of AIBN as a thermal initiator, 5.0 wt.% of PETA as a cross-linking agent, and 50 μL of FEC additive. Then, 20 μL of this solution was injected into the 3D ceramic framework thrice, followed by heating at 70 °C for 10 h in an oven to facilitate the occurrence of the in-situ polymerization reaction. For comparative purposes, the solid polymer electrolyte (SPE) was synthesized using a similar procedure to that of the CSE; however, Whatman® glass fiber was employed in place of the 3D ceramic framework.

1.4. Material characterization

The crystal structure of the synthesized materials was investigated using an X-ray diffractometer (XRD; Empyrean, Malvern Panalytical) with Bragg–Brentano geometry under the accelerating electron conditions of 40 kV and 30 mA at the Cu anode (λ = 1.5406 Å), and the XRD patterns were recorded using a PIXcel^{3D} detector at an angular resolution of 0.026°

and measurement time per step of 46 s. The XRD patterns were analyzed using the Rietveld refinement method in the X'Pert Highscore Plus software environment.[4] A Fourier transform infrared spectrometer equipped with the attenuated total reflection (ATR) mode (FTIR; Spectrum 400, PerkinElmer) was used to identify the molecular structures of the composite materials. The Raman spectra of these materials were obtained using a Laser Raman spectroscope (NRS-5100, JASCO). The surface chemistry of the composite materials was analyzed using a high-performance X-ray photoelectron spectrometer (HP–XPS; K–Alpha^{+}, Thermo Scientific) equipped with a micro-focused X-ray source. Binding energies were calibrated against the C 1 s peak at 284.8 eV. The obtained spectra were interpreted using the Gaussian–Lorentzian distribution. The morphology of the prepared samples was investigated using a field emission scanning electron microscope (FESEM; Gemini 500, Zeiss) operated at an accelerating voltage of 15 kV and equipped with a 50 mm^2 energy-dispersive X-ray detector (EDS; ATW2, Oxford). The cross-section SEM specimens were milled using an argon-ion beam in a cooling cross-section polisher (CCP; IB-19520CCP, Jeol) operated in the intermittent milling mode, and the process was cooled to a temperature of –30 °C by using liquid nitrogen to avoid thermal damage to the specimens. ^{6}Li direct-polarisation magic-angle spinning solid-state nuclear magnetic resonance (MAR-SSNMR) analysis was performed using an ECZ400R spectrometer (Jeol) at 9.4 T (400 MHz) with a ^{6}Li Larmor frequency of 58.8 MHz. The samples were packed in a 4 mm zirconia rotor and spun at a speed of 10 kHz. The 90° pulse length was 10 μs, and the recycle delay was 10 s. Moreover, 256 scans were acquired for all samples. The percentage conversion of monomers was measured by ^{1}H NMR analysis (AVANCE III HD 400, Bruker) using dimethyl sulfoxide-d6 as a solvent. Gel Permeation Chromatography (GPC) was used to determine the molecular weight of SPE. The thermal behavior of the composites was characterized using a thermogravimetric analyzer (TGA-50, Shimadzu).

1.5. Electrochemical characterization

Electrochemical characterization was performed using CR2032-type coin cells, which were assembled in an Ar- filled glove box. The ionic conductivity (*σ*) of the CSE in a symmetric cell was measured using **Equation 1** and the electrochemical impedance spectroscopy (EIS) technique in an electrochemical workstation (Zive SP2). The symmetric cells were fabricated by placing an inorganic ceramic film on a stainless-steel (SS) disc, after which the monomer solution was injected following the above procedure. Another SS disc was inserted, and then, the CR2032 cells were assembled. Finally, the assembled cells were treated in an oven at 70 °C for 10 h.

$$\sigma = \frac{t}{RS} \quad (1)$$

where *t* is the thickness, *S* is the interaction surface area of the CSE, and *R* is its resistance.

According to **Equation 2**, the activation energy (E_a) of the CSEs is calculated from the slope of the log *σ* *vs.* 1/*T* plot, as follows:

$$\sigma = Ae^{\frac{-E_a}{RT}} \quad (2)$$

where *σ* is the ionic conductivity, *A* is a pre-exponential constant, *R* is the universal gas constant, and *T* is the temperature.

The Li|3D-LLZT-CSE|SS asymmetric cells were prepared by following the same above process, except one of the two SS discs was replaced with a 16 mm lithium chip. The electrochemical potential stability of the CSEs was evaluated using the linear sweep voltammetry (LSV) technique in an electrochemical workstation (Zive SP2) from the open circuit potential to 6 V *vs.* Li/Li^+ at a scan rate of 1 mV s^{-1} at 30 °C.

Additionally, the Li-ion transference numbers (t_{Li^+}) of the CSEs were calculated with the Bruce and Vincent method,[5] which is expressed as **Equation 3**, by using a single-step chronoamperometry technique with an overpotential of 10 mV at 30 °C applied to the Li|3D-LLZT-CSE|Li lithium symmetric cells.

$$t_{Li^+} = \frac{I_{ss}}{I_o} \times \frac{V - I_o R_o}{V - I_{ss} R_{ss}} \quad (3)$$

where I_o and R_o denote the current and resistance before polarization, respectively; I_{ss} and R_{ss} are the steady-state current and resistance after polarization, respectively; and ΔV is the applied overpotential.

The Li^+ plating/stripping behavior of CSE in the Li|3D-LLZT-CSE|Li symmetric cell was measured to investigate the stability of the CSE in the presence of Li metal by using a battery cycler (WonATech-WBCS 3000).

The cathode materials (NCM811 or NVMP@C) were blended with Super P carbon (Timcal) and 2 wt.% PVDF or 2 wt.% PAA in NMP solvent as a binder in a weight ratio of 7:2:1 to obtain a homogeneous slurry. Thereafter, the slurry was cast onto an Al foil as a current collector by using a doctor blade. Then, the slurry was dried in a vacuum oven at 80 °C for 12 h. Subsequently, circular electrodes measuring 14 mm in diameter were punched from the foil. The loading amount of the cathode material was controlled to approximately 1.0–1.5 mg cm^{-2} in the subsequent electrochemical characterization.

To fabricate lithium-ion batteries, a lithium chip measuring 16 mm in diameter and punched from a lithium foil was covered with 10 μL of the monomer precursor solution and used as the anode. NCM811 permeated with 10 μL of the electrolyte precursor solution was used as the cathode after the electrolyte fully penetrated its pores. Then, we fabricated the Li|3D-LLZT-CSE|NCM811 cells by following the same procedure as that for symmetric cells. For comparison, the Li||NCM811 cell was fabricated using 75 μL of 1 M $LiPF_6$ in EC/DEC (50:50 vol.%) as the liquid electrolyte and Whatman® glass fiber as the separator.

To prepare the sodium-ion batteries, we started by wiping the kerosene off Na cubes stored in kerosene (99.8% purity, Sigma-Aldrich). Then, we cut these cubes into smaller cubes and pressed them to obtain approximately 150-μm-thick Na foils. Then, discs measuring 16 mm were punched from these foils and used as anodes. To confirm the performance of as-

synthesized NVMP@C, a Na||NVMP@C cell was fabricated using 75 μL of 0.6 M $NaPF_6$ in EC/DMC (30:70 vol.%) with 5 vol.% FEC additive as the liquid electrolyte and Whatman® glass fibre as the separator. Furthermore, the Na|3D-NZLSP-CSE|NVMP@C cells were fabricated by following a similar procedure as that for Li|3D-LLZT-CSE|NCM811 with 3D-NZLSP as the inorganic framework.

A single-layer pouch cell was constructed utilizing a cathode material (NCM811 or NVMP@C) measuring 2.5 cm × 3.5 cm, with a loading mass of 2 – 2.5 mg cm^{-2}, a 3D-ceramic framework, and an anode material (Li or Na foil). Subsequently, 350 μL of the monomer solution was introduced into the assembly, which was then vacuum-sealed within a laminated aluminum film inside a glove box to obtain the pouch cell. Finally, the pouch cell was heated to initiate polymerization of the monomer.

Galvanostatic charge-discharge experiments were conducted using an automatic battery cycler (WBCS 3000, WonATech) in the cell potential ranges of 2.5–4.3 V for the Li|3D-LLZT-CSE|NCM811 cells and 2.5–4.0 V for the Na|3D-NZLSP-CSE|NVMP@C cells. The C-rates of the NCM811 and NVMP@C cathodes were calculated based on the specific capacities of 200 mAh g^{-1} and 117.6 mAh g^{-1}, respectively, which corresponds to 1 C. The obtained specific capacity was calculated based on the masses of the cathode materials. All electrochemical characterizations were performed in a temperature chamber at 30 °C.

1.6. Computational method

The density-functional-theory (DFT) calculations were implemented using an ultra-soft pseudo-potential and the generalized gradient approximation in the Heyd–Scuseria–Ernzerhof exchange-correlation functional (HSE) in Quantum Espresso package.[6] The structures were relaxed using the Broyden–Fletcher–Goldfarb–Shanno (BFGS) algorithm with force and energy convergences of less than 10^{-5} Ry/Bohr and 10^{-5} Ry, respectively. The nudged elastic band (NEB) method was used to calculate the Li migration barrier. The electrostatic potential

maps and natural bond orbital (NBO) charge were calculated using Gaussian 09. The VESTA and XcrySDen programs were used for visualization.[7]

2. Tables

Table S1. Rietveld refinement results for the nominal composition of 3D-LLZT framework

Structure	Space group	Lattice constant [Å]	Agreement factors [%]			
		a = b = c	R_{exp}	R_p	R_{wp}	χ^2
Cubic	$Ia\bar{3}d$	12.92305	5.00716	4.34001	5.63697	1.26739

Atom	Wyckoff site	x	y	z	Occupation
Li(1)	96h	0.062	0.35	0.0.321	0.293
Li(2)	24d	0.375	0	0.25	0.96
La	24c	0.125	0	0.25	1
Zr	16a	0	0	0	0.7
Ta	16a	0	0	0	0.3
O	96h	0.1017	0.1962	0.2816	1

Table S2. Rietveld refinement results for nominal composition of NCM811

Structure	Space group	Lattice constant [Å]		Agreement factors [%]			
		a = b	c	R_{exp}	R_p	R_{wp}	χ^2
Cubic	$R\bar{3}m$	2.87075	14.19747	4.76863	3.37124	4.99405	1.09678

Atom	Wyckoff site	x	y	z	Occupation
Li	3b	0	0	0.5	1.0
Ni	3a	0	0	0	0.8
Co	3a	0	0	0	0.1
Mn	3a	0	0	0	0.1
O	6c	0	0	0.26637	1.0

Table S3. Comparison of the electrochemical performances of solid electrolytes when using NCM cathode at room temperature

**Config**	**C-rate and corresponding specific capacity** [mAh g^{-1}]								
	0.1 C	0.2 C	0.3 C	0.5 C	1 C	2 C	3 C	4 C	5 C
Li|PTFE-LLZTO-SN|NCM523[8]	158	–	148	130	–	–	–	–	–
Li|PAN-insitu|NCM622[9]	173.1	–	126.2	51	–	–	–	–	–
Li|QSPE|NMC811[10]	–	172	–	161	144	114	–	71	–
Li|DSPE-2M|NMC811[11]	168	165	–	153	137	118	–	–	–
Li|FRSE|NCM811[12]	210.2	199.1	–	182.1	169.6	115.8	–	–	–
This work	180.3	170	–	150.7	133.2	108.8	–	–	65.5

Table S4. Quantification of Li^+ local environment in 3D-LLZT-CSE before and after polarisation

**Sample**		**Li^+ local environment**			**Total**
		LLZT	Polymer	Interphase	
Before	Area	1350.10	731.61	105.72	2187.43
	Percent [%]	61.72	33.45	4.83	100
After	Area	3793.19	5950.05	1333.89	11077.13
	Percent [%]	34.22	53.69	12.09	100
Increase factor		2.8	8.1	12.6	5.1

Table S5. Rietveld refinement results obtained for nominal composition of 3D-NZLSP

Agreement factors [%]			
R_{exp}	R_p	R_{wp}	χ^2
9.12636	10.44547	14.85024	2.64772

Phase	Atom	Wyckoff site	x	y	z	Occupation
**$Na_3Zr_2(SiO_4)_2(PO_4)$** (79.1%)	Na(1)	4d	0.25	0.25	0.5	0.81
	Na(2)	4e	0.5	0.891	0.25	1
	Na(3)	8f	0.836	0.079	0.842	0.6
	Zr	8f	0.1015	0.2472	0.0539	1
	Si(1)	4e	0	0.0392	0.25	0.67
	P(1)	4e	0	0.0392	0.25	0.33
	Si(2)	8f	0.3569	0.1117	0.2586	0.67
	P(2)	8f	0.3569	0.1117	0.2586	0.33
	O(1)	8f	0.1466	0.4366	0.2226	1
	O(2)	8f	0.4374	0.4439	0.0821	1
	O(3)	8f	0.2527	0.1812	0.2028	1
	O(4)	8f	0.3812	0.1337	0.1116	1
	O(5)	8f	0.4491	0.1809	0.4369	1
	O(6)	8f	0.0806	0.1475	0.2444	1

Structure	Space group	Lattice constant			
		a [Å]	b [Å]	c [Å]	β [°]
Monoclinic	C2/c	15.7465	9.1211	9.1750	124.5507

Phase	Atom	Wyckoff site	x	y	z	Occupation
**$Na_3La(PO_4)_2$** (20.9%)	Na(1)	4a	0.1164	0.5	0.44935	1
	Na(2)	4a	0.0498	0.0003	0.35021	1
	Na(3)	4a	0.0316	0.5305	0.10931	1
	Na(4)	4a	0.2018	0.495	0.26891	1
	Na(5)	4a	0.6337	0.002	0.17061	1
	Na(6)	4a	0.2774	0.0013	0.00881	1
	La(1)	4a	0.88329	0.04942	0.19666	1
	La(2)	4a	0.36648	0.53793	0.42276	1
	P(1)	4a	0.26741	0.0119	0.34571	1
	P(2)	4a	0.27188	0.4707	0.10174	1

P(3)	4a	0.48245	0.4809	0.27406	1
P(4)	4a	0.02057	0.0227	0.0177	1
O(1)	4a	0.3747	0.5132	0.27381	1
O(2)	4a	0.616	0.0984	0.00281	1
O(3)	4a	0.198	0.5976	0.14671	1
O(4)	4a	0.4987	0.0119	0.09761	1
O(5)	4a	0.0292	0.2984	0	1
O(6)	4a	0.0166	0.5219	0.35181	1
O(7)	4a	0.5116	0.2352	0.23701	1
O(8)	4a	0.2388	0.2497	0.38151	1
O(9)	4a	0.0277	0.2928	0.23481	1
O(10)	4a	0.2333	0.0081	0.26821	1
O(11)	4a	0.7241	0.204	0.38681	1
O(12)	4a	0.2497	0.4863	0.02201	1
O(13)	4a	0.2775	0.1928	0.12244	1
O(14)	4a	0.0537	0.0922	0.47251	1
O(15)	4a	0.8755	0.0143	0.34461	1
O(16)	4a	0.3635	0.6038	0.11611	1

**Structure**	**Space group**	**Lattice constant** [Å]		
		a	b	c
Orthorhombic	$Pca2_1$	14.09932	5.35035	18.70834

Table S6. Rietveld refinement results obtained for nominal composition of $Na_3V_{1.95}Mg_{0.05}(PO_4)_3$@C

**Structure**	**Space group**	**Lattice constant** [Å]		**Agreement factors** [%]			
		a = b	c	R_{exp}	R_p	R_{wp}	χ^2
Trigonal	$R\bar{3}c$	8.7288	21.8042	4.46344	5.11115	6.73935	2.2798

**Atom**	**Wyckoff site**	**x**	**y**	**z**	**Occupation**
Na(1)	6b	0	0	0	0.805
Na(2)	18e	0.63397	0	0.25	0.731
V(1)	12c	0	0	0.14717	0.975
Mg	12c	0	0	0.14717	0.025
P(1)	18e	0.29061	0	0.25	1
O(1)	36f	0.1914	0.16902	0.08905	1
O(2)	36f	0.029	0.20714	0.1931	1

Table S7. Comparison of electrochemical performances of solid electrolytes and NaSICON at room temperature

**Config**	**C-rate and corresponding specific capacity** [mAh g^{-1}]													**Cyclability** [cycles | capacity | C-rate]
	0.1 C	0.2 C	0.3 C	0.5 C	1 C	2 C	3 C	4 C	5 C	6 C	8 C	10 C	15 C	
Na|ETPTA-$NaClO_4$|NVP@C[13]	–	–	–	–	103	–	–	–	–	88	–	75	55	1000 | 98 mAh g^{-1} | 1 C
Na|NZCSP|NVP@C[14]	–	103.1	101.1	100.5	96.7	90.3	–	80.5	–	–	–	–	–	500 | 94.9 mAh g^{-1} | 1 C
Na|PGT32-5%|NVP[15]	102.9	100	–	95.9	92.3	86.3	80	–	–	–	–	–	–	400 | 92.8 mAh g^{-1} | 0.2 C
Na|DSPE-20|NVP@C[16]	–	104.1	–	103.2	102	98.9	97.9		93.5	–	–	–	–	300 | 96.1 mAh g^{-1} | 1 C
Na|NZLSP-IL|NVP[2]	–	113	–	112	109	106	–	103	–	97	91	86	–	10000 | 90 mAh g^{-1} | 10 C
Na|GPE|NVP[17]	–	–	–	93	93	90	87	–	79	–	–	–	–	1000 | 98 mAh g^{-1} | 1 C
This work (PAA binder)	109.7	108.7	–	103.7	99.2	94.7	–	–	85.3	–	–	76.7	–	3000 | 95 mAh g^{-1} | 2 C
This work (PVDF binder)	100.2	95.9	–	89.3	82.6	75.7	–	–	53.9	–	–	0.7	–	–

3. Supporting Videos

Because the size of videos is too large and exceeds the maximum upload file size of 40 MB, we provide the Google Drive link for each video

Supporting Video 1: Link

Supporting Video 2: Link

Supporting Video 3: Link

Supporting Video 4: Link

4. Figures

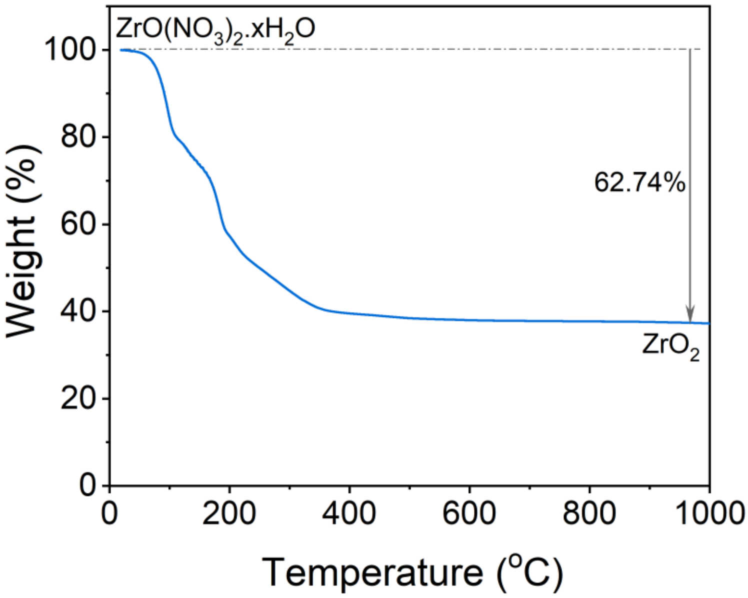

Figure S1. TGA curve of $ZrO(NO_3)_2.xH_2O$ in air atmosphere.

Figure S1 illustrates the TGA curve of $ZrO(NO_3)_2.xH_2O$ in air atmosphere in the temperature range of 25−1000 °C. $ZrO(NO_3)_2.xH_2O$ decomposed and released H_2O, O_2, and NO_2 to ZrO_2 according to the following chain reaction:

$$ZrO(NO_3)_2.xH_2O \longrightarrow ZrO(NO_3)_2 + xH_2O\uparrow \longrightarrow ZrO + O_2\uparrow + NO_2\uparrow \longrightarrow ZrO_2$$

Therefore, the degree of hydration was calculated as follows:

$$x = \frac{\frac{\frac{100}{wt\%_{1000^oC}}}{M_{ZrO_2}} - M_{ZrO(NO_3)_2}}{M_{H_2O}} \tag{4}$$

where M_{ZrO_2}, $M_{ZrO(NO_3)_2}$, and M_{H_2O} are the molecular masses of ZrO_2, $ZrO(NO_3)_2.xH_2O$, and H_2O, respectively. $wt.\%_{1000^oC}$ is the weight percentage of the residual samples at 1000°C. The calculated degree of hydration is 5.52, meaning that the nominal chemical formula of the precursor is $ZrO(NO_3)_2{\cdot}(5.52H_2O)$.

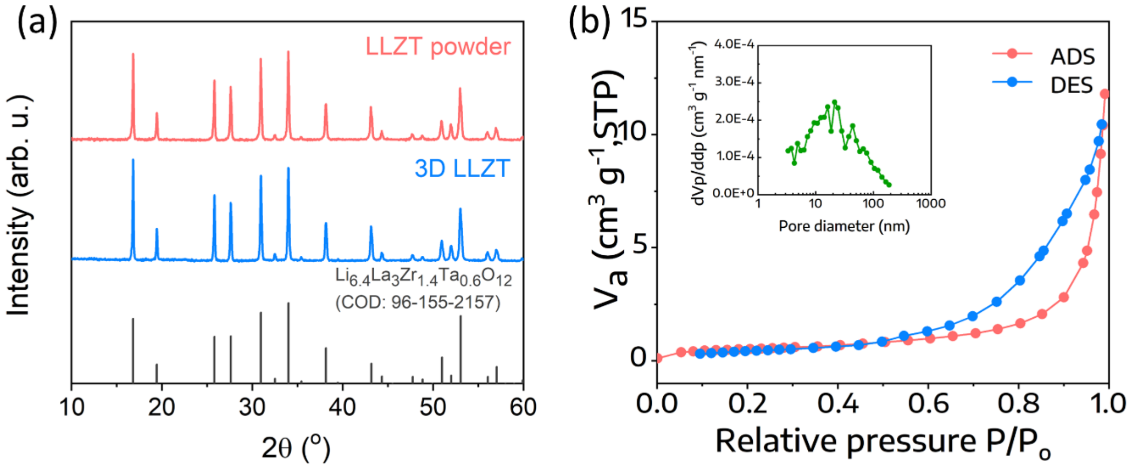

Figure S2. (a) XRD patterns of LLZT powder and 3D-LLZT framework. (b) BET analysis results for 3D-LLZT. The BJH plot of the pore-size distribution is shown in the inset.

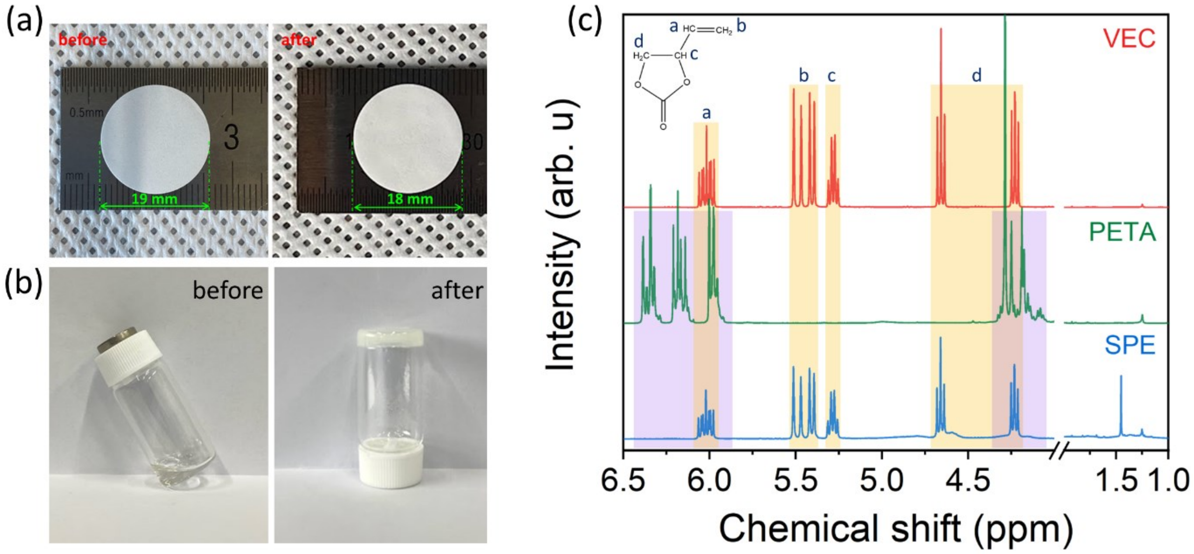

Figure S3. (a) Photographs of the 3D-LLZT framework before and after sintering, and (b) photographs of the monomer solution before and after polymerization. (c) ^{1}H NMR spectra of VEC, PETA, and SPE.

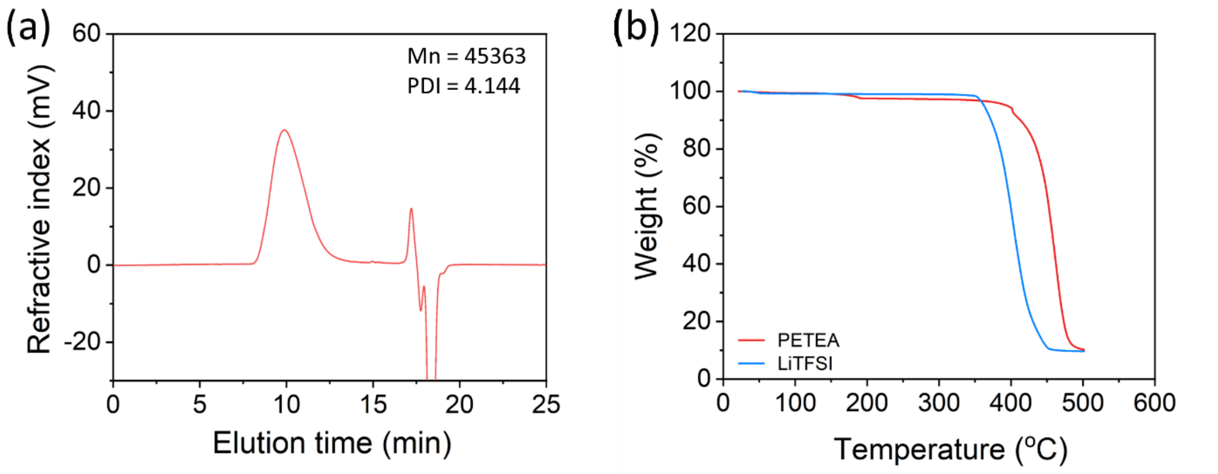

Figure S4. (a) GPC result of SPE. (b) TGA curves of PETA and LiTFSI in air atmosphere.

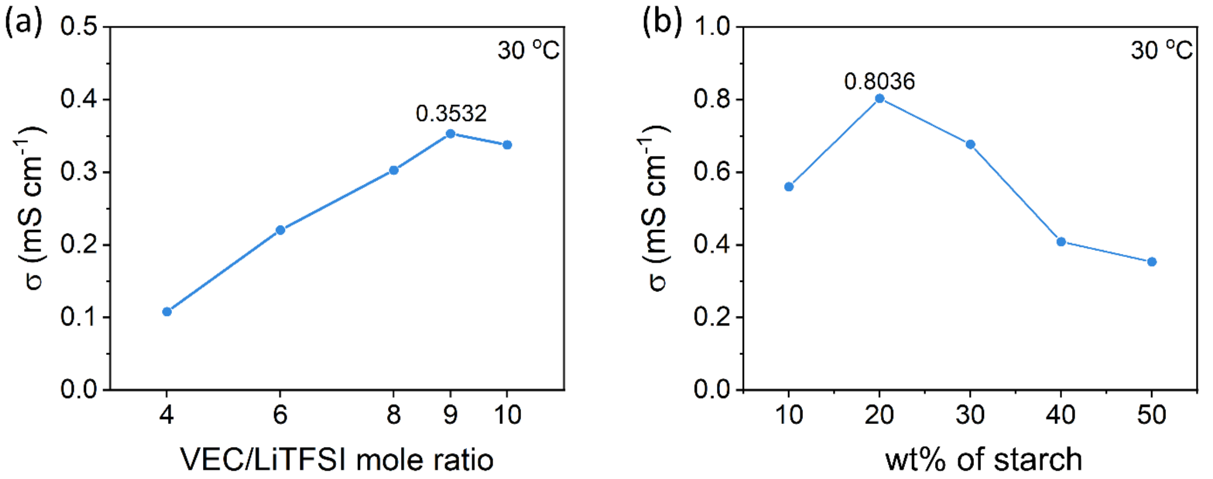

Figure S5. Ionic conductivity of 3D-LLZT-CSE as a function of (a) VEC/LiTFSI mole ratio and (b) wt.% of starch at 30 °C.

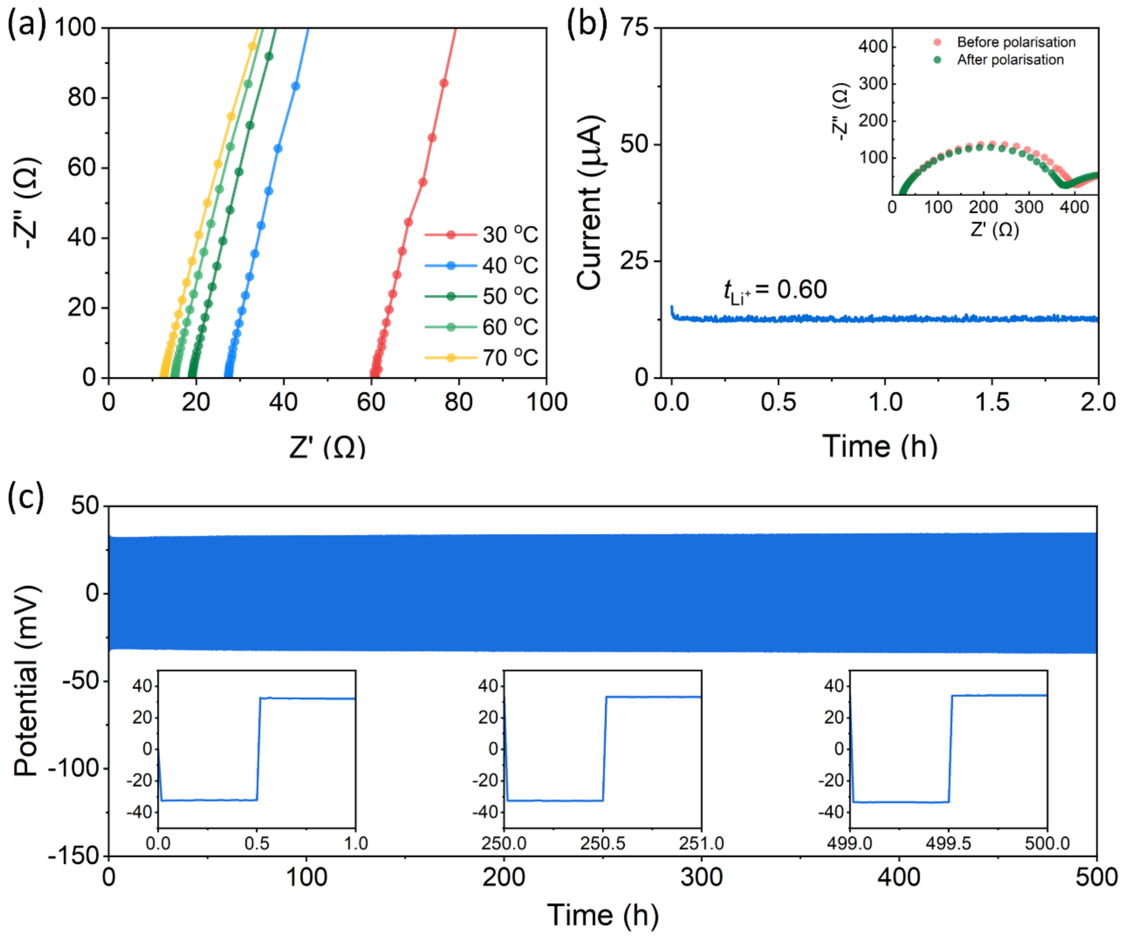

Figure S6. (a) Nyquist plots for SPE at different temperatures. (b) Current transient profile and the corresponding EIS plots of Li|SPE|Li symmetric cell before and after polarization. (c) Cyclability of the Li|3D-LLZT-CSE|Li symmetric cell at a current density of 1.0 mA cm^{-2} and a temperature of 30 °C.

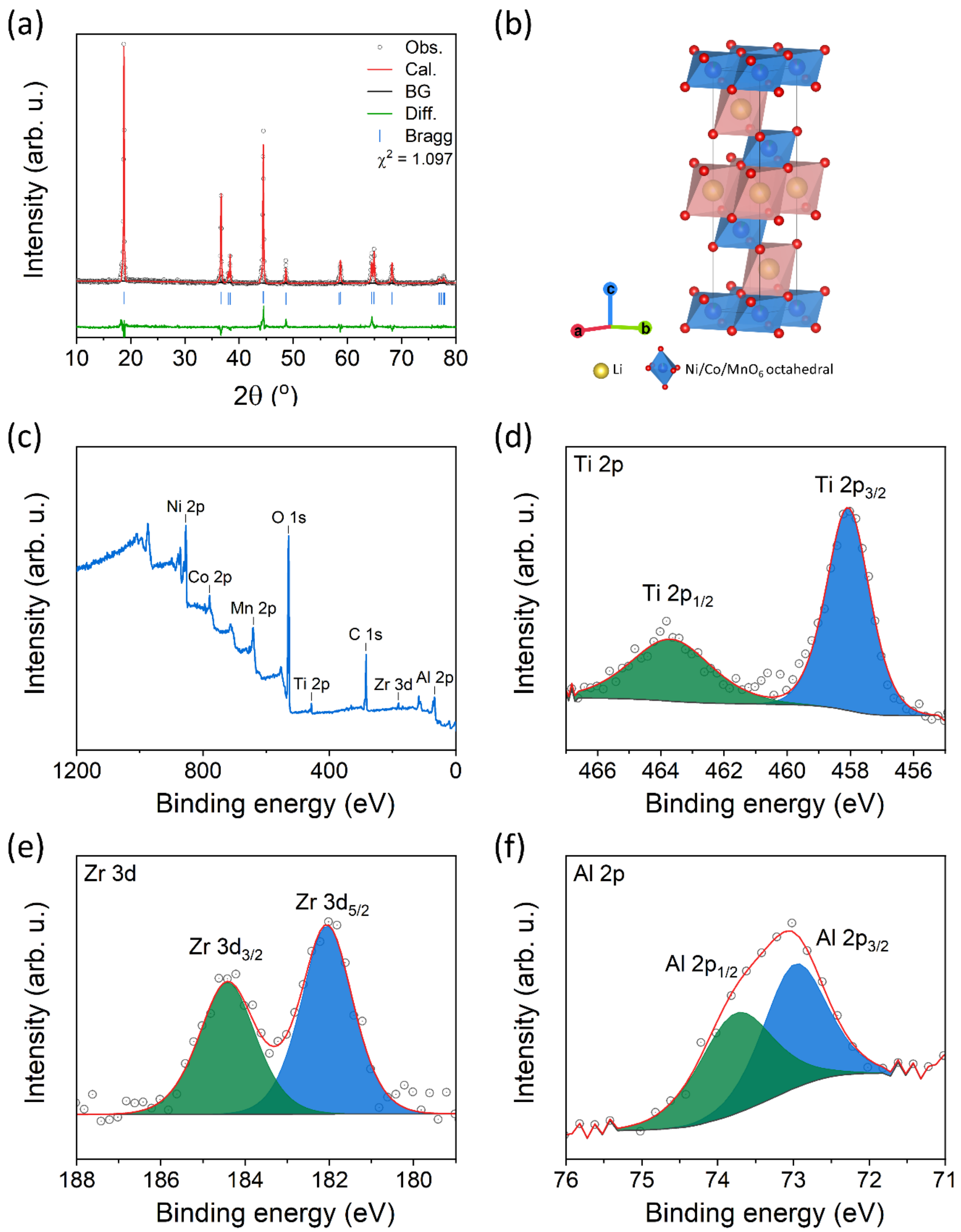

Figure S7. (a) Rietveld-refined XRD pattern and (b) crystal structure of NCM811. (c) Wide-range XPS spectrum and high-resolution XPS spectra of (d) Ti 2p, (e) Zr 3d, (f) Al 2p in NCM811 material.

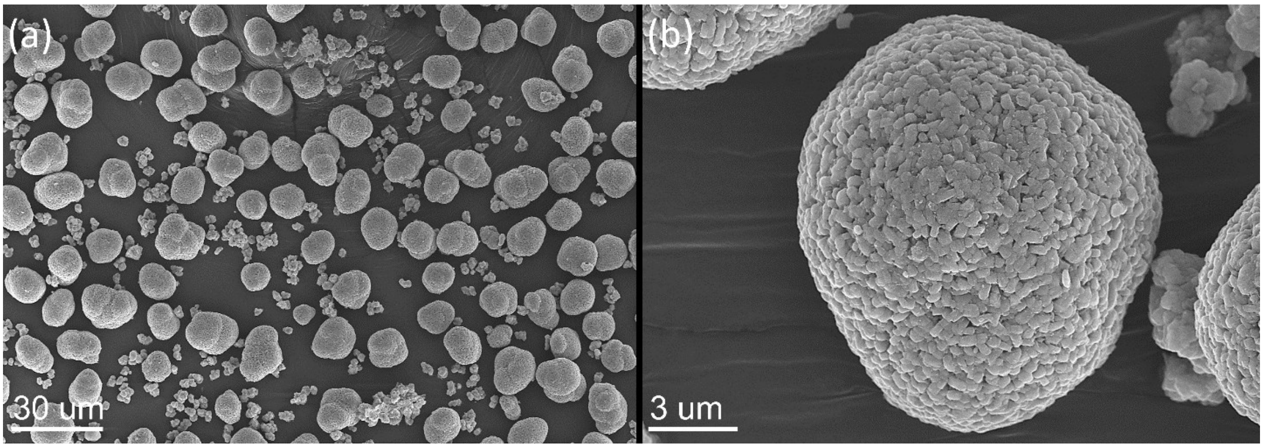

Figure S8. SEM images of NCM811 material at (a) low and (b) high magnification.

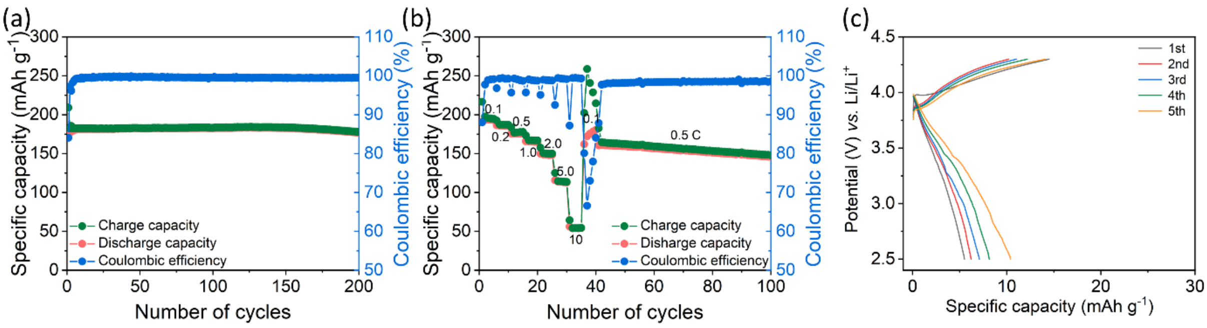

Figure S9. (a) Cyclability curve at 0.5 C and (b) rate capability of NCM811 cathode when using 1 M $LiPF_6$ in EC/DEC (50:50 vol.%) as the liquid electrolyte. (c) Galvanostatic charge-discharge profiles of Li|SPE|NCM811 at 0.1 C.

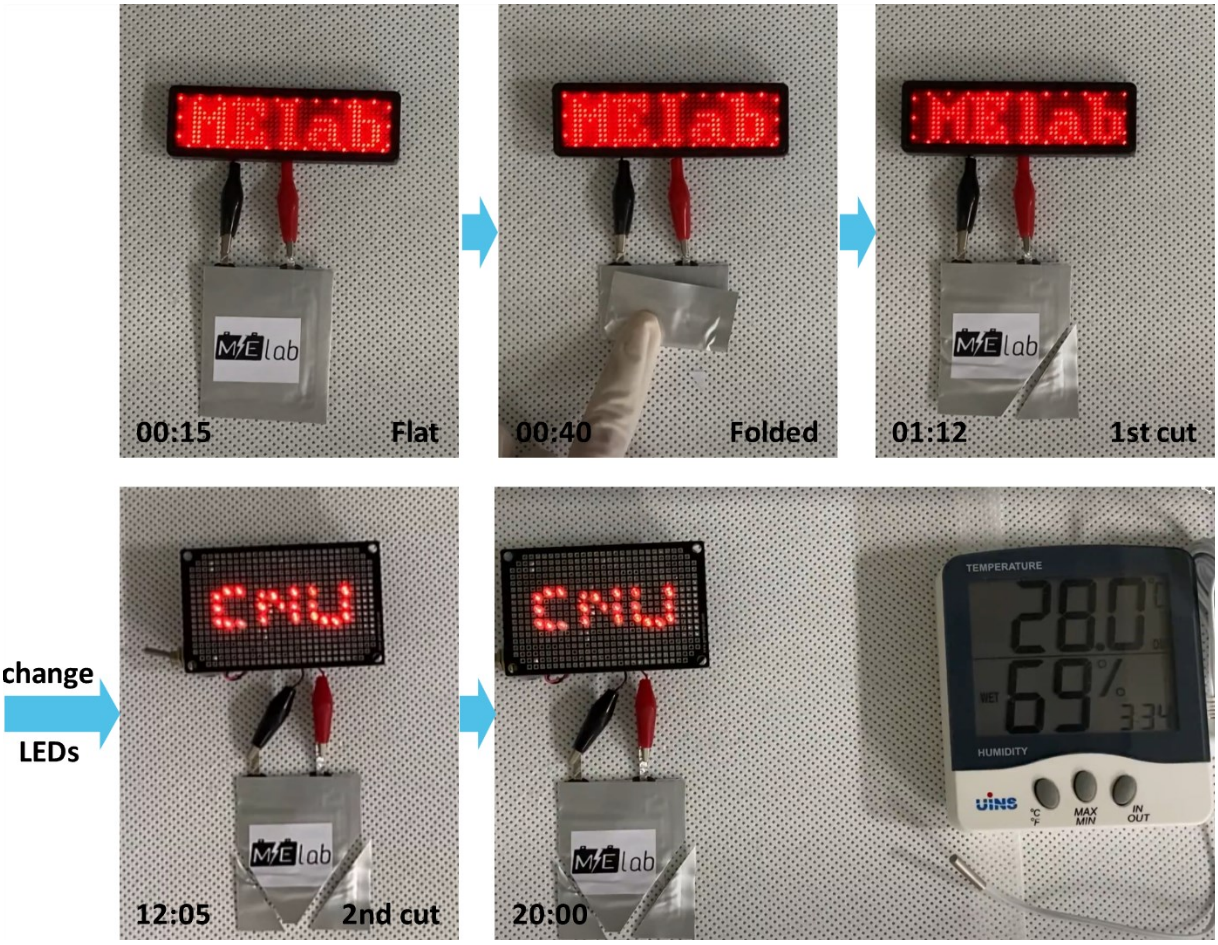

Figure S10. Photographs of Li|3D-LLZT-CSE|NCM811 pouch cell that lights up the LED in the flat, folded, cut conditions with time.

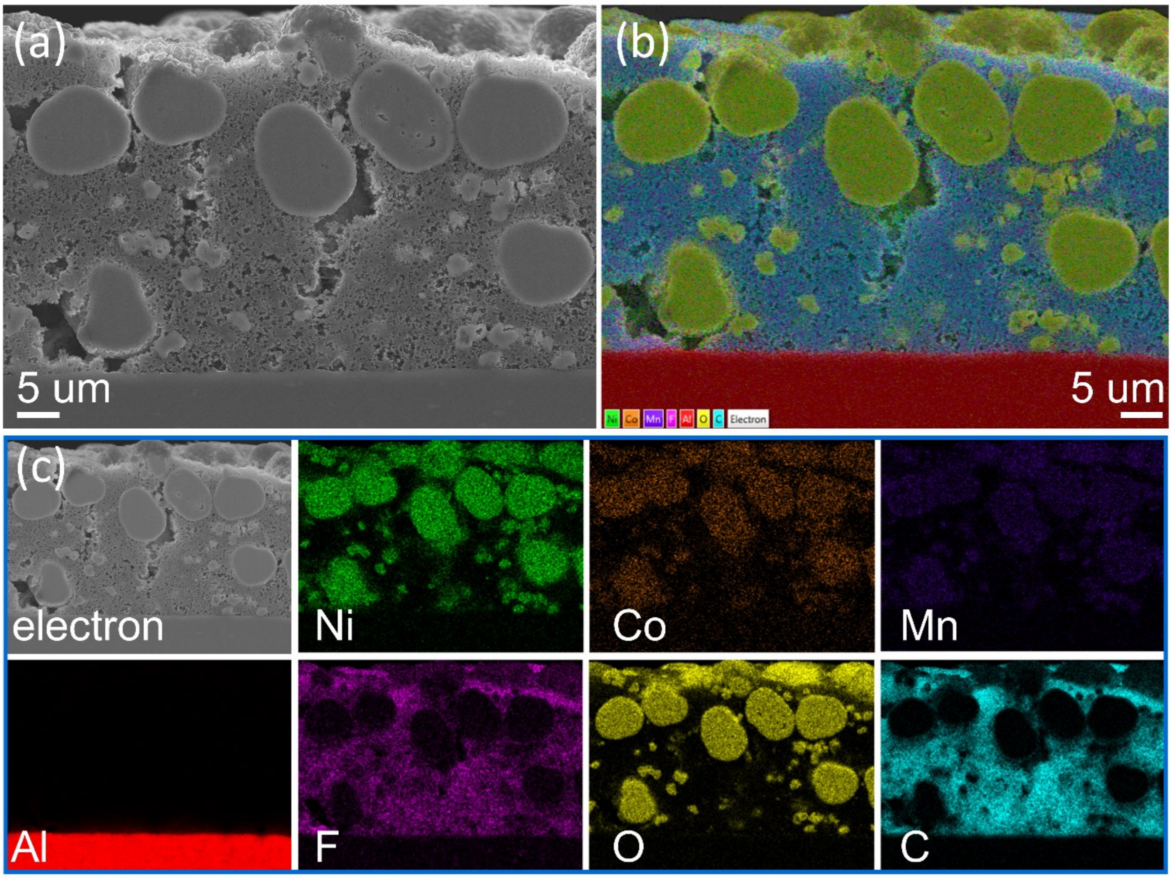

Figure S11. (a) Cross-sectional SEM image and (b, c) EDS mapping image of bare NCM811 electrode

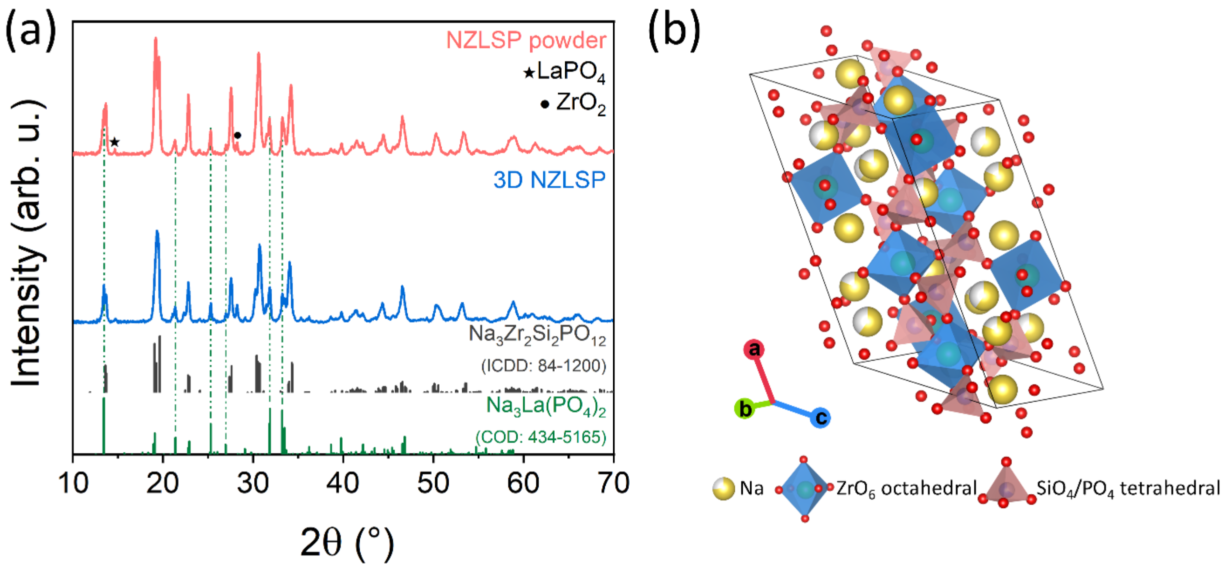

Figure S12. XRD patterns of NZLSP powder and 3D-NZLSP framework. (b) Crystal structure of 3D-NZLSP.

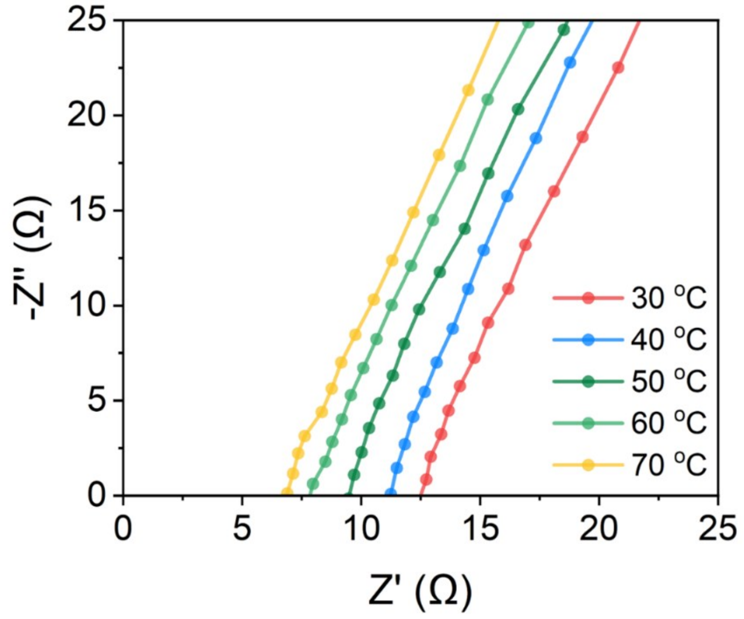

Figure S13. Nyquist plots of 3D-NZLSP-CSE at different temperatures.

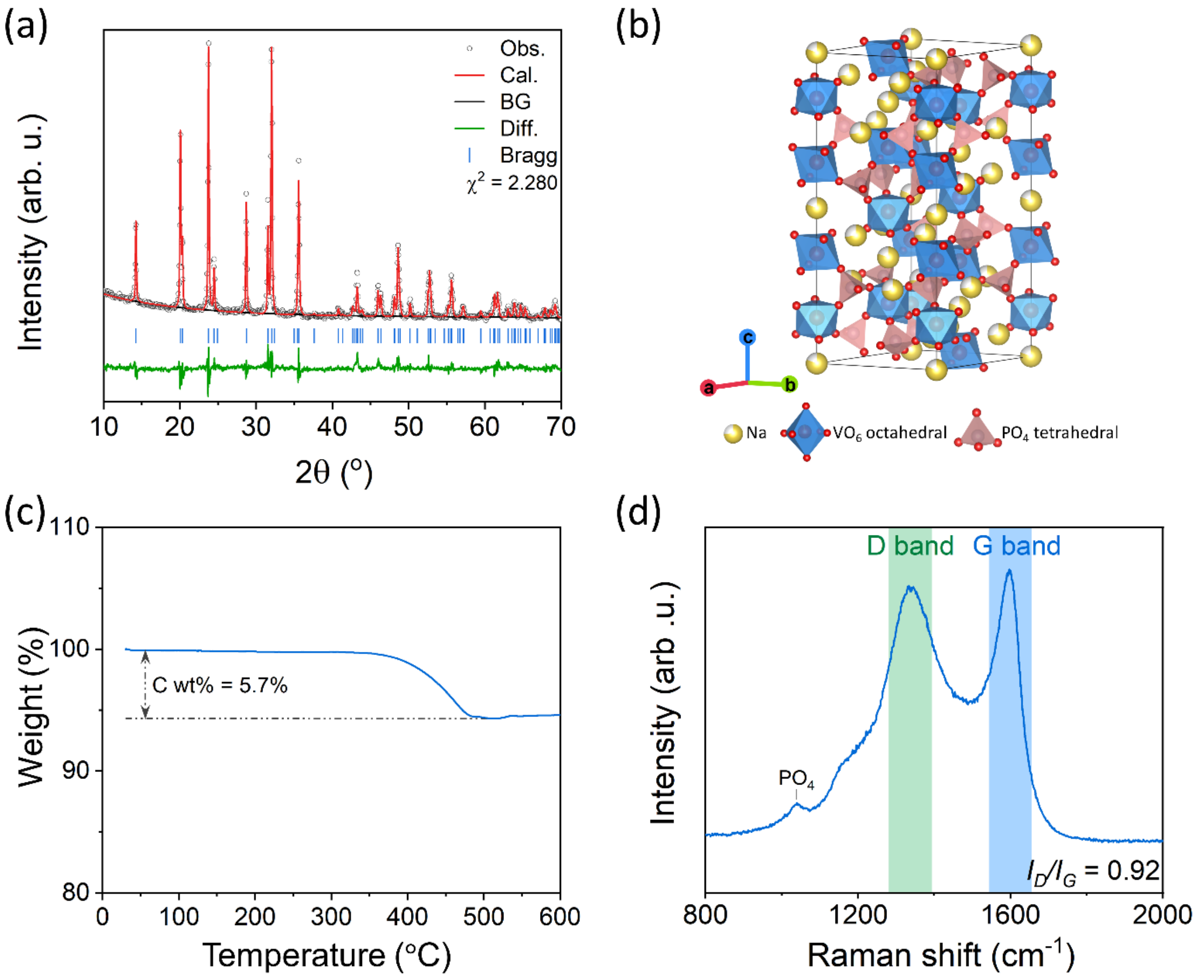

Figure S14. (a) Rietveld-refined XRD pattern and (b) crystal structure. (c) TGA curve in air atmosphere. (d) Raman spectrum of NVMP@C cathode.

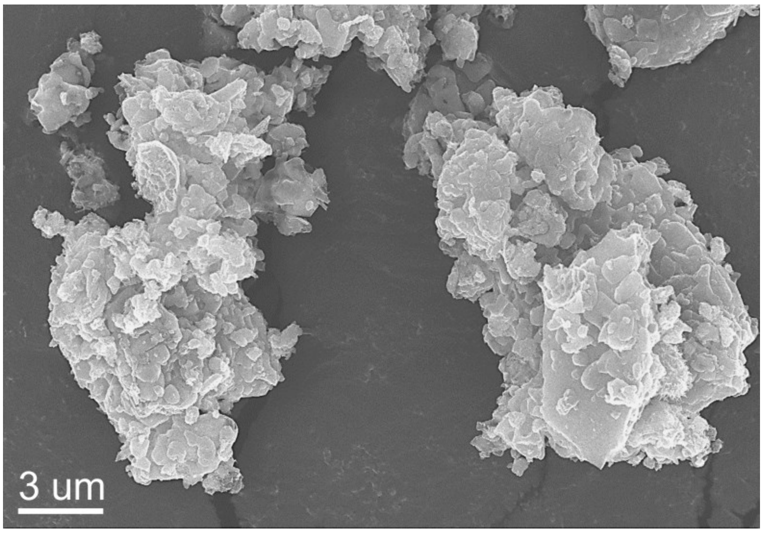

Figure S15. SEM image of NVMP@C material.

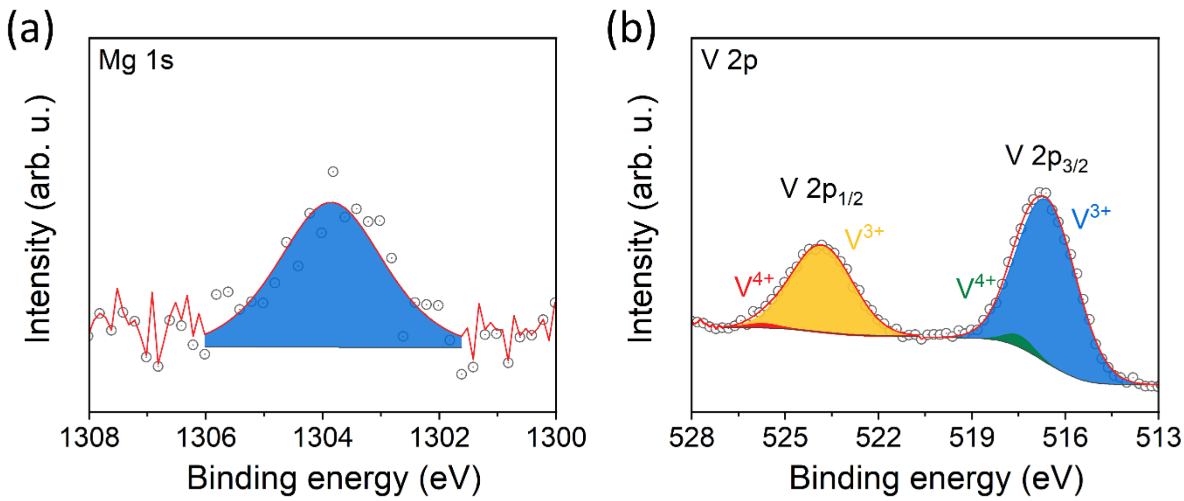

Figure S16. High-resolution XPS spectra of (a) Mg 1s and (b) V 2p of NVMP@C material.

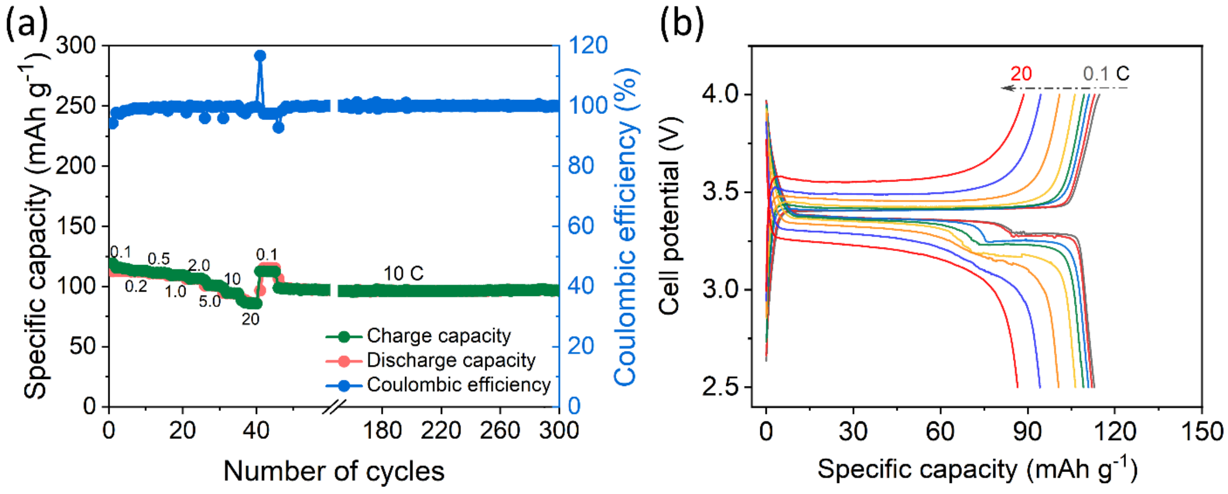

Figure S17. (a) Rate capability and (b) corresponding galvanostatic charge-discharge profiles of NVMP@C cathode at different C-rates when using 0.6 M $NaPF_6$ in EC/DMC (30:70 vol.%) with 5 vol.% FEC additive as liquid electrolyte.

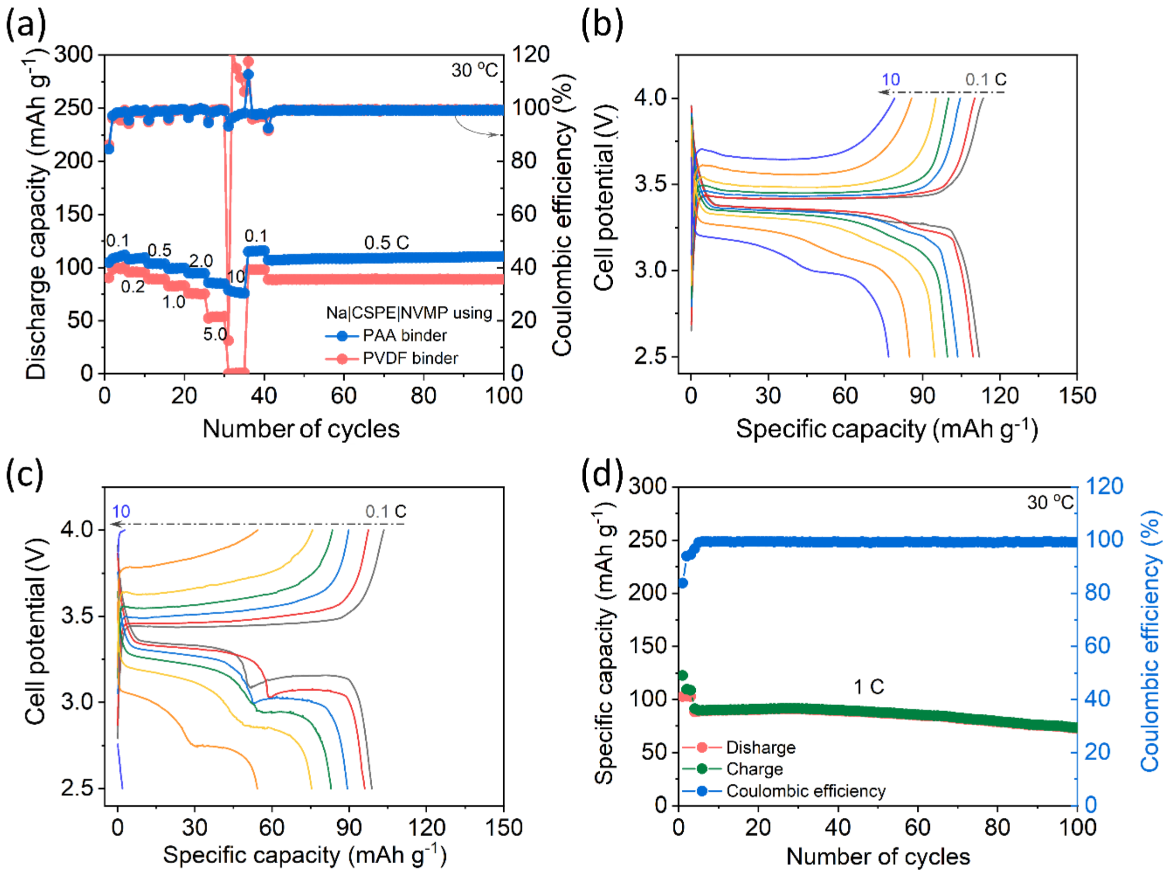

Figure S18. (a) Rate capability comparison of Na|3D-NZLSP-CSE|NVMP@C cell and (b, c) their corresponding galvanostatic charge-discharge profiles at different C-rates when using PAA and PVDF binders, respectively. (d) Cylability of Na|3D-NZLSP-CSE|NVMP@C pouch cell at 30 °C

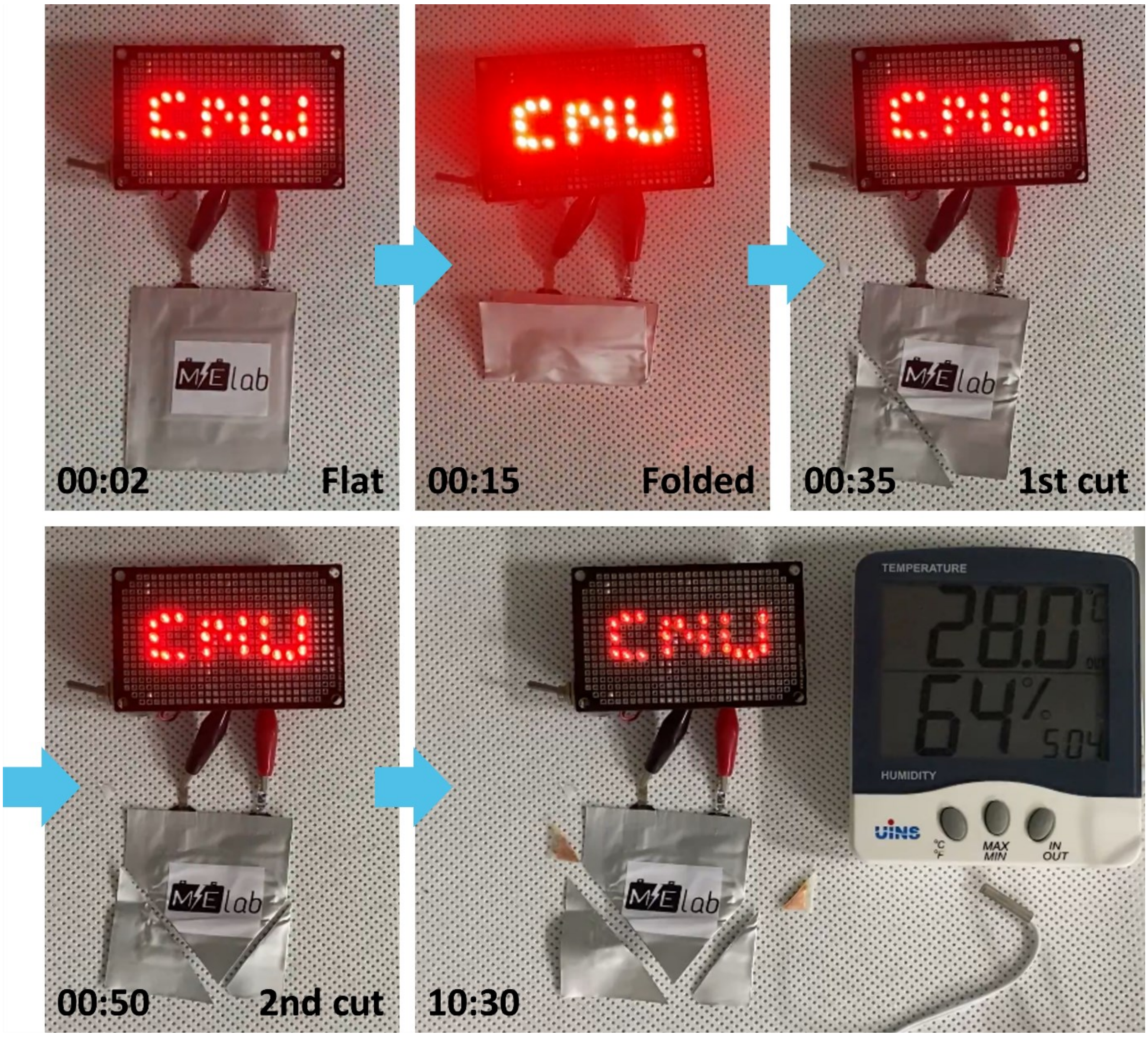

Figure S19. Photographs of Na|3D-NZLSP-CSE|NVMP@C pouch cell that lights up the LED in the flat, folded, cut conditions with time.

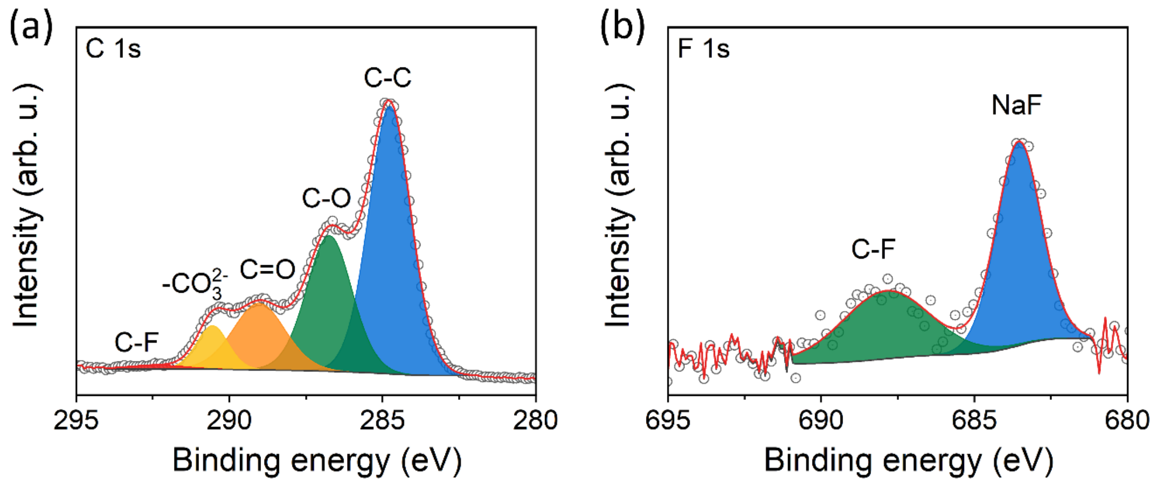

Figure S20. High-resolution XPS spectra of (a) C 1s and (b) F 1s of Na electrode after cycling test.

References

[1] K. Ishiguro, H. Nemori, S. Sunahiro, Y. Nakata, R. Sudo, M. Matsui, Y. Takeda, O. Yamamoto, N. Imanishi, *J. Electrochem. Soc.* **2014**, 161, A668.

[2] Z. Z. Zhang, Q. H. Zhang, J. A. Shi, Y. S. Chu, X. Q. Yu, K. Q. Xu, M. Y. Ge, H. F. Yan, W. J. Li, L. Gu, Y. S. Hu, H. Li, X. Q. Yang, L. Q. Chen, X. J. Huang, *Adv. Energy Mater.* **2017**, 7.

[3] H. Li, X. Q. Yu, Y. Bai, F. Wu, C. Wu, L. Y. Liu, X. Q. Yang, *J. Mater. Chem. A* **2015**, 3, 9578.

[4] H. M. Rietveld, *J. Appl. Crystallogr.* **1969**.

[5] E. J, A. C, Vincent., G. P, Bruce., *Polymer* **1987**, 28, 2324.

[6] a) P. Giannozzi, O. Andreussi, T. Brumme, O. Bunau, M. B. Nardelli, M. Calandra, R. Car, C. Cavazzoni, D. Ceresoli, M. Cococcioni, N. Colonna, I. Carnimeo, A. Dal Corso, S. de Gironcoli, P. Delugas, R. A. DiStasio, A. Ferretti, A. Floris, G. Fratesi, G. Fugallo, R. Gebauer, U. Gerstmann, F. Giustino, T. Gorni, J. Jia, M. Kawamura, H. Y. Ko, A. Kokalj, E. Kucukbenli, M. Lazzeri, M. Marsili, N. Marzari, F. Mauri, N. L. Nguyen, H. V. Nguyen, A. Otero-de-la-Roza, L. Paulatto, S. Ponce, D. Rocca, R. Sabatini, B. Santra, M. Schlipf, A. P. Seitsonen, A. Smogunov, I. Timrov, T. Thonhauser, P. Umari, N. Vast, X. Wu, S. Baroni, *J. Phys-Condens. Mat.* **2017**, 29;

b) P. Giannozzi, S. Baroni, N. Bonini, M. Calandra, R. Car, C. Cavazzoni, D. Ceresoli, G. L. Chiarotti, M. Cococcioni, I. Dabo, A. Dal Corso, S. de Gironcoli, S. Fabris, G. Fratesi, R. Gebauer, U. Gerstmann, C. Gougoussis, A. Kokalj, M. Lazzeri, L. Martin-Samos, N. Marzari, F. Mauri, R. Mazzarello, S. Paolini, A. Pasquarello, L. Paulatto, C. Sbraccia, S. Scandolo, G. Sclauzero, A. P. Seitsonen, A. Smogunov, P. Umari, R. M. Wentzcovitch, *J. Phys-Condens. Mat.* **2009**, 21;

c) P. Giannozzi, O. Baseggio, P. Bonfa, D. Brunato, R. Car, I. Carnimeo, C. Cavazzoni, S. de Gironcoli, P. Delugas, F. F. Ruffino, A. Ferretti, N. Marzari, I. Timrov, A. Urru, S. Baroni, *J. Chem. Phys.* **2020**, 152.

[7] a) A. Kokalj, *J. Mol. Graph. Model.* **1999**, 17, 176;

b) K. Momma, F. Izumi, *J. Appl. Crystallogr.* **2011**, 44, 1272.

[8] T. L. Jiang, P. G. He, G. X. Wang, Y. Shen, C. W. Nan, L. Z. Fan, *Adv. Energy. Mater.* **2020**, 10.

[9] M. Yao, Q. Q. Ruan, T. H. Yu, H. T. Zhang, S. J. Zhang, *Energy Storage Mater.* **2022**, 44, 93.

[10] J. Yu, X. D. Lin, J. P. Liu, J. T. T. Yu, M. J. Robson, G. D. Zhou, H. M. Law, H. R. Wang, B. Z. Tang, F. Ciucci, *Adv. Energy Mater.* **2022**, 12.

[11] X. D. Lin, J. Yu, M. B. Effat, G. D. Zhou, M. J. Robson, S. C. T. Kwok, H. J. Li, S. Y. Zhan, Y. L. Shang, F. Ciucci, *Adv. Funct. Mater.* **2021**, 31.

[12] S. J. Tan, J. P. Yue, Y. F. Tian, Q. Ma, J. Wan, Y. Xiao, J. Zhang, Y. X. Yin, R. Wen, S. Xin, Y. G. Guo, *Energy Storage Mater.* **2021**, 39, 186.

[13] P. C. Wen, P. F. Lu, X. Y. Shi, Y. Yao, H. D. Shi, H. Q. Liu, Y. Yu, Z. S. Wu, *Adv. Energy Mater.* **2021**, 11.

[14] Y. Lu, J. A. Alonso, Q. Yi, L. Lu, Z. L. Wang, C. W. Sun, *Adv. Energy Mater.* **2019**, 9.

[15] G. H. Chen, K. Zhang, Y. R. Liu, L. Ye, Y. S. Gao, W. R. Lin, H. J. Xu, X. R. Wang, Y. Bai, C. Wu, *Chem. Eng. J.* **2020**, 401.

[16] H. M. Law, J. Yu, S. C. T. Kwot, G. D. Zhou, M. Robson, J. X. Wu, F. Ciucci, *Energy Storage Mater.* **2022**, 46, 182.

[17] W. C. Zhang, J. Zhang, X. C. Liu, H. Li, Y. Guo, C. N. Geng, Y. Tao, Q. H. Yang, *Adv. Funct. Mater.* **2022**, 32.
